# Genome-Wide Identification and Evolution-Profiling Analysis of *Heat Shock Protein* Gene Family in *Poaceae Barnhart*

**DOI:** 10.3390/ijms26094269

**Published:** 2025-04-30

**Authors:** Xiaoyi Huang, Yue Liu, Xiao Yu, Yajun Cai, Lingyu Hou, Jingyuan Zhang, Hongchun Yang

**Affiliations:** 1 State Key Laboratory of Hybrid Rice, College of Life Sciences, Wuhan University, Wuhan 430072, China; xiaoyih@whu.edu.cn (X.H.); 17837184182@163.com (X.Y.); yajuncai@whu.edu.cn (Y.C.); zhangjy_cls@whu.edu.cn (J.Z.); 2 Institute of Applied Ecology, Chinese Academy of Sciences, Shenyang 110866, China; yueliu@iae.ac.cn; 3 School of Environmental Studies, China University of Geosciences (Wuhan), Wuhan 430078, China; hurly@cug.edu.cn

**Keywords:** *Poaceae*, cold stress, gene duplication events, HSP

## Abstract

Heat shock proteins (HSPs) function as molecular chaperones to maintain protein homeostasis and repair denatured proteins, counteracting abiotic stresses. Despite their functional importance, a systematic bioinformatics analysis of the *HSP* gene family was lacking in *Poaceae*. In this study, we revealed that HSPs are widely distributed from algae to eudicots, with varying numbers in *Poaceae* including *Oryza*, *Triticum*, and *Panicum*. Gene duplication events, particularly dispersed duplication (DSD), tandem duplication (TD), and genome polyploidization, have probably driven the increased number of *HSP* genes and the expansion of HSP family proteins. Gene Ontology (GO) annotation analyses suggested their conserved functions. Promoter cis-acting element analyses indicated that their expression levels were tightly regulated by abiotic stresses. We validated that many collinear *HSP* genes are indeed regulated by the cold stress by analyzing the published RNA-seq data in rice, maize, and wheat, and performing RT-qPCR in rice. Our findings shed light on the role of HSPs in the abiotic stress response, laying the groundwork for further exploration of HSP functions in *Poaceae*.

## 1. Introduction

Plants are facing increasingly severe abiotic stresses such as extreme temperatures, ionic stress, drought, and soil salinity [1,2,3,4,5]. As one of the major abiotic stresses, cold stress suppresses cell division, photosynthesis, and nutrient assimilation [6,7,8]. Plants have evolved many tolerance mechanisms to counteract these adverse effects [9]. Heat shock proteins (HSPs) act as molecular chaperones, participating in protein folding, stabilization, and repair, and prevent the aggregation of misfolded proteins during environmental stresses [10,11,12,13].

HSPs initially identified as being induced by heat shock [14], which is now known to be triggered by a wide range of external stresses [15,16,17]. Based on their molecular weight, sequence homology, and functional specificity, HSPs are categorized into five classes: HSP20, HSP60, HSP70, HSP90, and HSP100 [18,19]. These chaperones localize to many subcellular compartments, including the cytoplasm, endoplasmic reticulum, chloroplasts, mitochondria, and nucleus, enabling stress responses in these compartments [13]. The HSP20 family is the most genetically diverse molecular chaperones, and is induced by low or high temperatures [16,20,21,22]. The expression of *CsHSP17.5* changes seasonally in chestnut, peaking in winter, coinciding with enhanced plant cold resistance. It is likely involved in low-temperature adaptation [23]. A large number of HSP70 proteins are present in eukaryotes. Their expression is induced by various stresses such as drought [24], high salt [25], heavy metals [26], and pathogen infection [27]. *HSP70-14/15*-silenced *Arabidopsis* plants are more sensitive to heat treatment, displaying a reduced survival rate under high temperatures and poor recovery after heat stress [28]. HSP90 plays a pivotal role in immune systems [29,30]. Overexpressing *TaHSP90.2* and *TaHSP90.3* leads to enhanced resistance to stripe rust in wheat [31]. In rice, the Hop/Sti1-HSP90 complex is involved in chitin-triggered immunity and fungal immunity [32]. Given their widespread roles in plant responses to biotic and abiotic stresses [33], studying the functions of HSPs has great significance.

HSP families are widely distributed in various species, including *Arabidopsis* [34,35,36], rice [37,38,39], and wheat [40,41,42]. AtHSP90 regulates root and hypocotyl development by stabilizing key proteins in the auxin signaling pathway, such as TRANSPORT INHIBITOR RESPONSE 1 (TIR1) and PIN-FORMED 1 (PIN 1). Knockout mutants of *HSP90.7* show downregulated auxin synthesis pathways, abnormal root hairs, and chloroplast function [43]. Inhibiting AtHSP90 activity leads to morphological variations in leaves [44]. These findings highlight the critical roles of AtHSP90 family members in plant growth and development, particularly in maintaining auxin homeostasis and regulating organ development. The overexpression of *OsHSP101* enhances heat acclimation and forms a positive feedback loop with HSA32. This loop prolongs the effect of heat acclimation, improving long-term acquired thermotolerance. This mechanism is conserved across plant species and is crucial for the heat stress response in rice [45]. However, the identification and evolutionary relationships of HSPs in *Poaceae Barnhart* (*Poaceae*) remain incomplete. Several species of *Poaceae* plants are important economic crops, which have high-quality genome sequences for investigating the evolutionary relationships of protein families [46,47]. In this study, we systematically identified members of the HSP20, HSP60, HSP70, and HSP90 families in *Oryza*, *Triticum,* and *Panicum* plants. We conducted a systematic bioinformatics analysis to reveal their phylogenetic relationships and duplication events. The functional annotation/prediction and cis-acting element analysis of these *HSP* genes suggested that their expression is dynamically regulated and they potentially participate in many processes besides stress response. Additionally, we analyzed the expression patterns of collinear genes under cold treatment in *OsHSPs*, *ZmHSPs*, and *TaHSPs* using RNA-seq data. The RT-qPCR results showed dynamic expression patterns for randomly selected collinear *OsHSP* genes, suggesting they may play a role in cold response. These findings provide useful information for understanding the evolutionary relationships and biological functions of HSP families in *Poaceae*.

## 2. Results

### 2.1. The Number of HSPs Is Increased in Higher Plants

**Figure 1:**
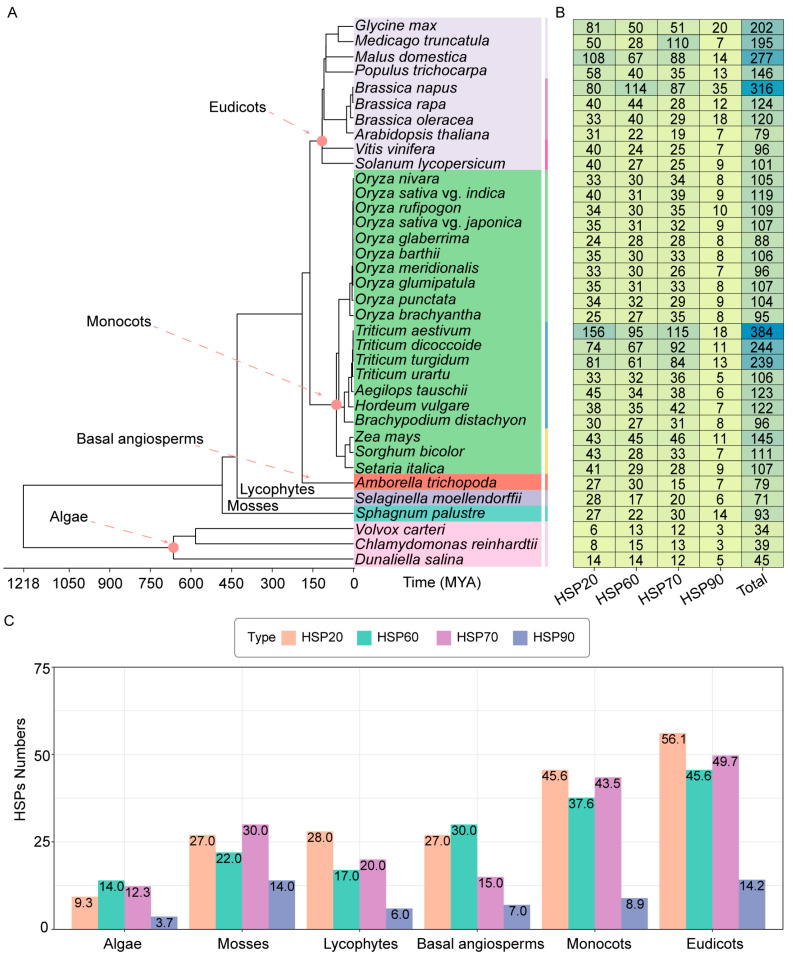
The number of HSP members in representative species during evolution. (**A**) Phylogenetic tree of the selected 36 representative species. The time scale shows the evolution of species. MYA strand for millions of years ago. The evolutionary tree was constructed by the maximum likelihood method (bootstrap values: 1000 replicates) using RaxML. The tree was visualized using the R package ggtree v3.2.1. (**B**) The heat map shows the number of HSP20, HSP60, HSP70, and HSP90 protein members, as well as the total number of HSPs for each species. A darker shade of blue indicates a higher abundance of HSPs. (**C**) The average number of HSPs in different groups. Three algae species (*Volvox carteri*, *Chlamydomonas reinhardtii*, and *Dunaliella salina*), one moss species (*Sphagnum palustre*), one lycophyte species (*Selaginella moellendorffii*), one basal angiosperm species (*Amborella trichopoda*), twenty monocot species, and ten eudicot species were used to identify the number of HSPs. The horizontal axis indicates the plant taxa, and the vertical axis indicates the number of HSPs. Different colors are used to indicate the types of HSPs.

To explore the distribution of HSPs in plants, we systematically identified HSPs from 36 representative species including algae, bryophytes, lycophytes, basal angiosperms, monocots, and eudicots (Figure 1A). The amino sequences of HSPs were retrieved from the Ensembl Plants, Phytozome 13, and WheatGene databases. The longest protein sequence for each protein was used for the phylogenetic analysis. In 36 representative species, a total of 4730 HSPs were identified, comprising 1583 HSP20, 1320 HSP60, 1468 HSP70, and 359 HSP90 proteins. BLAST 2.9.0+ software alignment and Hidden Markov model (HMM) screening were used to analyze and visualize the phylogenetic evolution (Supplemental Data S1). We found that the number of HSPs gradually increased from aquatic plants to land plants. The number of HSPs was relatively low in algae (34–45). In mosses, lycophytes, and basal angiosperms, the number of HSPs (71–93) increased significantly (Figure 1B). In rice, the number of HSPs ranged from 88 to 119, suggesting a diversity compared to algae lycophytes, and basal angiosperms. The minimal difference in the number of HSPs between cultivated (88–119) and wild rice (95–109) indicated that they have maintained a high degree of similarity in HSP evolution. In wheat, the number of HSPs was significantly increased in *Triticum aestivum* (allohexaploid), *Triticum turgidum* (tetraploid), and *Triticum dicoccoide* (tetraploid) compared to that in diploid wheat, suggesting that genome duplication through hybridization probably is the main cause. In *Panicum*, maize (145 HSPs) has more HSPs than sorghum (111 HSPs) and millet (107 HSPs). In eudicots, the distribution of HSPs showed a gradually increasing trend. In *Solanum lycopersicum* and *Vitis vinifera*, the number of HSPs was relatively consistent (101 and 96, respectively). In *Brassicaceae*, the number of HSPs showed an increase (ranging from 79 to 316). In *legumes*, such as soybean and medicago, the distribution of HSP20, HSP60, HSP70, and HSP90 protein members also showed significant differences. These results indicate that compared to monocots, the distribution of HSP members in eudicots plants is more complex.

### 2.2. Phylogenetic Analysis of HSPs in Poaceae

The average number of HSPs in each plant group was calculated. In three kinds of algae (*Volvox carteri*, *Chlamydomonas reinhardtii*, and *Dunaliella salina*), the average numbers of HSPs were 9 HSP20, 14 HSP60, 12 HSP70, and 4 HSP90 proteins. In mosses (*Phagnum palustre*), these numbers were 27, 22, 30, and 14, respectively. In lycophytes (*Selaginella moellendorffii*), they were 28, 17, 20, and 6. In basal angiosperms (*Amborella trichopoda*), the numbers were 27, 30, 15, and 7. In 20 representative monocots, the average numbers of HSPs were 46 HSP20, 38 HSP60, 44 HSP70, and 9 HSP90 proteins. In 10 representative eudicots, the averages were 56 HSP20, 46 HSP60, 50 HSP70, and 14 HSP90 proteins (Figure 1C). HSP20, HSP60, and HSP70 proteins are more abundant in monocots and eudicots, possibly aiding in responses to environmental stresses. Interestingly, HSP90 proteins have minimal variation in numbers, suggesting their conserved biological functions during stress responses.

HSPs were ubiquitously distributed from aquatic algae to terrestrial monocots and eudicots, indicating their evolutionary conservation. The numbers of HSPs in mosses, lycophytes, and basal angiosperms were increased compared to those in algae plants. As plants evolved from aquatic to terrestrial habitats, they encountered more biotic and abiotic stresses, which might explain the expansion of the *HSP* genes.

HSPs play a critical role in stress responses. When comparing different plant groups, *Poaceae* have more HSPs than algae, mosses, and basal angiosperms. This increase in HSPs in *Poaceae* suggests that they may have more diverse functions. We compared the evolutionary relationships among HSPs from different species in *Poaceae*, using the HSPs of the model species *Arabidopsis* as a reference.

**Figure 2:**
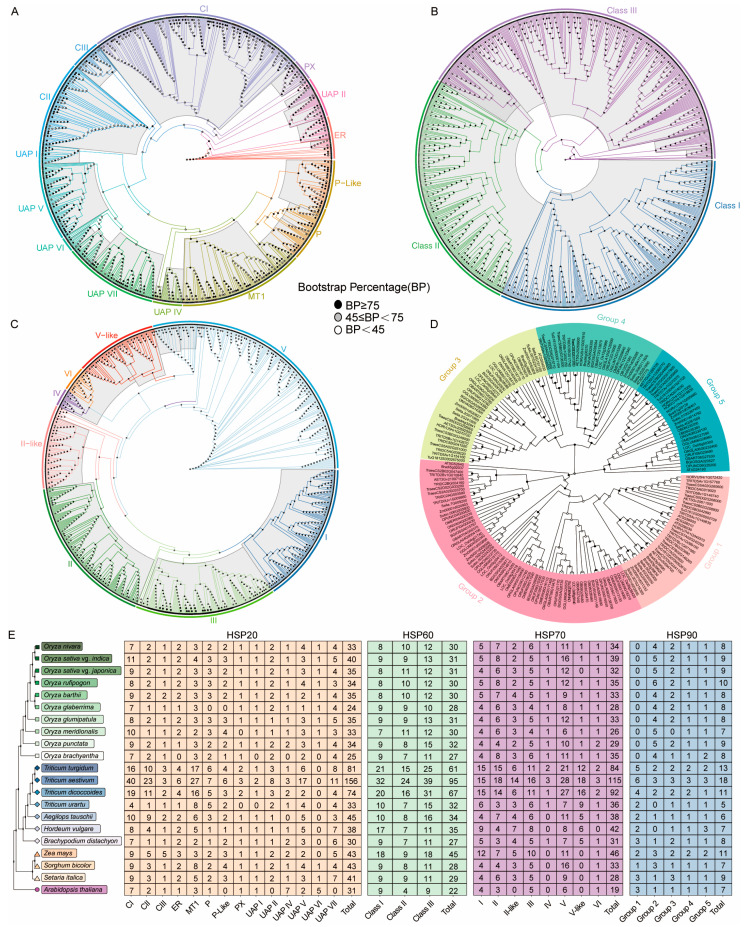
The phylogenetic relationships of HSP subfamilies in *Oryza*, *Triticum*, and *Panicum*. The phylogenetic relationships of HSP20 (**A**), HSP60 (**B**), HSP70 (**C**), and HSP90 (**D**) subfamilies in *Oryza*, *Triticum*, and *Panicum*. The maximum likelihood phylogenetic trees were constructed using RAxML-ng-v1.2.0 software with 1000 bootstraps. Different subfamilies are shown with different labels. The phylogenetic tree of 21 species on the left. *Arabidopsis*, *Oryza*, *Triticum*, and *Panicum* are shown in pink, green, blue, and yellow, respectively. (**E**) The rectangle chart shows the number of different subfamily members. From left to right are HSP20, HSP60, HSP70, and HSP90. Subfamily classification of HSP family members was performed based on the bootstrap values of the phylogenetic tree and species information. The subfamily numbers were visualized using R package pheatmap 1.0.12.

The phylogenetic tree revealed that, among the *Poaceae* species, the HSP20 proteins could be divided into 14 subfamilies: CI, CII, CIII, ER, MT1, P, P-like, PX, UAP I, UAP II, UAP IV, UAP V, UAP VI, and UAP VII (Figure 2A). The CI subfamily was the largest (223 members), followed by MT1 (130) and UAP VII (99) (Figure 2E). The UAP VI subfamily was absent in wheat and present in most rice species and millet, suggesting it may have been lost during wheat evolution. The P-like, UAP II, and UAP VII subfamilies were identified in all of the plants, indicating that these subfamilies may have been maintained during evolution in *Poaceae* (Figure 2A,E). The HSP60 proteins were divided into Class I, Class II, and Class III subfamilies (Figure 2B). Class III was the largest subfamily (319) among the HSP60 proteins, followed by Class I (249) and Class II (208) (Figure 2E). In rice, the number of HSP60 members was evenly distributed between cultivated and wild rice. The number of Class I members in maize is two times higher than that in *Sorghum* and *Setaria*, and the number of Class III members is also higher than that in *Sorghum* and *Setaria*, suggesting that HSP60 proteins have been amplified in maize during evolution (Figure 2B,E). HSP70 proteins can be divided into eight subfamilies: I, II, II-like, III, IV, V, V-like, and VI (Figure 2C). Subfamily V was the largest (258 members), followed by II (150) and III (138). Among *Panicum*, maize has a higher number of HSP70 proteins than *Sorghum* and *Setaria* (Figure 2E). Meanwhile, members of subfamily IV and V-like were only found in rice and wheat, suggesting that these two subfamilies evolved in rice and wheat after the divergence of millet. The HSP90 proteins were divided into Group 1, Group 2, Group 3, Group 4, and Group 5 (Figure 2D). Group 2 was the largest (63), followed by Group 3 (36) and Group 1 (33) (Figure 2E). Group 1 and Group 2 have relatively close evolutionary relationships, and Group 3 and Group 4 have relatively close evolutionary relationships. Group 1 was absent in rice, indicating that Group 1 was lost during evolution. Group 2 was more abundant in rice than in wheat and millet, suggesting that there are differences in the evolution of HSP90 proteins in *Poaceae* (Figure 2E).

### 2.3. Gene Duplication Events Lead to the Expansion of HSP Genes

Gene duplication events play a pivotal role in driving the expansion of gene families in plants [48]. Common gene duplication types include singleton, dispersed duplication (DSD), proximal duplication (PD), tandem duplication (TD), and whole-genome duplication (WGD) [48]. We employed MCScanX to analyze the types of gene duplication among HSPs across 21 species.

**Figure 3:**
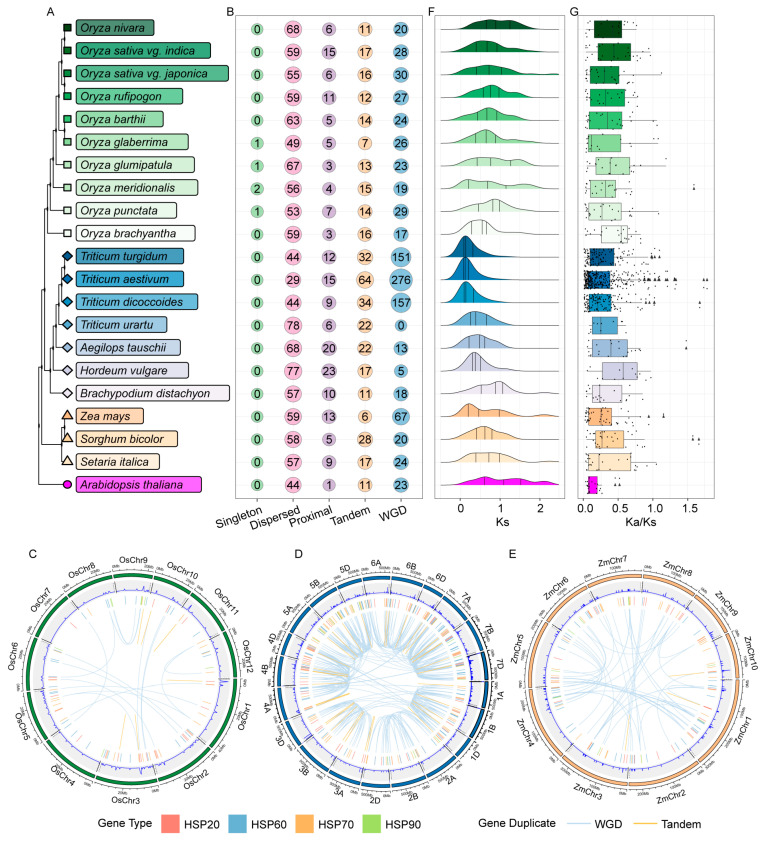
Duplication event analyses of *HSPs* in *Oryza*, *Triticum*, and *Panicum*. (**A**) Phylogenetic tree of HSPs among species. *Arabidopsis*, *Oryza*, *Triticum*, and *Panicum* are shown in pink, green, blue, and yellow, respectively. (**B**) The dot plots are used to show the duplication types of *HSP* genes. Singleton, dispersed duplication, proximal duplication, tandem duplication, and whole-genome duplication (WGD) are shown in green, pink, purple, yellow, and blue, respectively. The size of the dot indicates the number of duplication events of *HSPs*. MCScanX 0.8 software was used to analyze the gene duplication events. The duplicate_gene_classifier program was used to analyze the duplication type of genes. (**C**–**E**) The chromosome location and duplicated gene pairs of *HSPs* in *Oryza sativa np. Japonica* (**C**), *Triticum aestivum* (**D**), and *Zea mays* (**E**). The outer track is the physical size of each chromosome. The chromosomes of *Oryza*, *Triticum*, and *Zea mays* are shown in green, blue, and yellow, respectively. In the second track, line graphs show the number of HSP members in 500 Kb windows in each chromosome. In the third track, bar graphs indicate the gene type of HSPs. HSP20, HSP60, HSP70, and HSP90 are shown in red, blue, orange, and green, respectively. In the fourth track, curve graphs show the duplicated gene pairs. Whole-genome duplication (WGD) and tandem duplication (TD) events are shown in blue and orange, respectively. (**F**) The ridgeline charts are used to show the distribution of the Ks values of the duplicated gene pairs of *HSPs*. The three lines in the ridgeline charts indicate the first, second, and third quartile of Ks values. (**G**) The box plots show the distribution of the Ka/Ks values of the duplicated gene pairs of *HSPs*. The lower and upper ±1.5 quartiles are indicated by whiskers, the lower and upper ends of the boxes indicate the 25th and 75th quartiles, and the line across the middle of the box identifies the median sample value. The box length is the interquartile range (IQR), with upper and lower whiskers at 1.5 × IQR. The circular dots represent the Ka/Ks values, and the triangular dots represent the discrete values, which are all labeled on the central axis.

In rice, the majority of *HSP* genes undergo duplication via dispersed segmental duplication (DSD), followed by whole-genome duplication (WGD, or defined as polyploidization), and tandem duplication (TD). Except for the allopolyploid species *Triticum aestivum*, *Triticum turgidum*, and *Triticum dicoccoides*, the main type of gene duplication is DSD, followed by tandem duplication (TD) and proximal duplication (PD), similar to in rice. A proportion of 46.2% of the *HSP* genes in maize underwent WGD, followed by DSD and PD, while in *Sorghum* and *Setaria*, they underwent DSD (Figure 3B). Almost all *HSP* genes have undergone gene duplication, with DSD being the main cause of *HSP* gene expansion in *Oryza*, *Triticum*, and *Panicum* (Figure 3B). Overall, gene duplications play significant roles in evolution and increase the number of HSPs in these species.

TD and WGD are the most important mechanisms of gene expansion and gene family evolution [49,50]; we analyzed the chromosomal distribution and these two types of gene duplication events among species (Figure 2C–E). Based on the reference genome annotations, we analyzed the duplication events of *HSP* genes in rice, wheat, and maize. In *Oryza sativa*, we found 23 WGD pairs and 6 TD pairs (Figure 2C). In *Triticum aestivum*, each chromosome had distinct gene duplication types; notably, chromosomes 2A, 2B, and 2D had no TD (Figure 2D), indicating ancestral or gene structure differences among chromosomes. In *Zea mays*, there were 50 WGD pairs and 3 TD pairs, with WGD pairs being evenly distributed across chromosomes (Figure 2E). WGD likely provides abundant genetic material for evolution, promoting morphological and physiological diversity [51], while TD may help plants adapt to specific environments quickly [52].

The peak of Ks values can be used to assess changes in gene duplication during the evolution of gene families. The Ka/Ks ratio can be used to evaluate the evolutionary rate of genes. A higher Ka/Ks ratio (>1) may indicate that the gene has undergone significant functional changes during evolution, while a lower Ka/Ks ratio (<1) suggests that the gene has maintained conservative function [48].

In *Oryza*, the median Ks distribution is 0.54–0.89. In the allopolyploid species *Triticum aestivum*, *Triticum turgidum*, and *Triticum dicoccoides*, the median Ks is 0.11–0.13. In diploid wheat species, it is 0.35–0.47. In maize, sorghum, and foxtail millet, the median Ks values are 0.46, 0.61, and 0.72, respectively (Figure 3F). To further analyze whether these *HSP* gene pairs were influenced by selective pressures like purifying and positive selection, we calculated Ka/Ks values in *Poaceae*. The statistical results reveal that the Ka/Ks ratio shows a significant variation in *Poaceae* (Figure 3G). The average Ka/Ks ratio ranges from 0.29 to 0.54, which suggests that these genes have been highly conserved during evolution. Additionally, 32 gene pairs with Ka/Ks ratios greater than 1 were also identified, including 8 pairs in *Oryza*, 20 pairs in *Triticum*, and 4 pairs in *Panicum* (Figure 3G). These *HSP* gene pairs are inferred to have experienced positive selection during evolution, highlighting their key functional role in this process.

**Figure 4:**
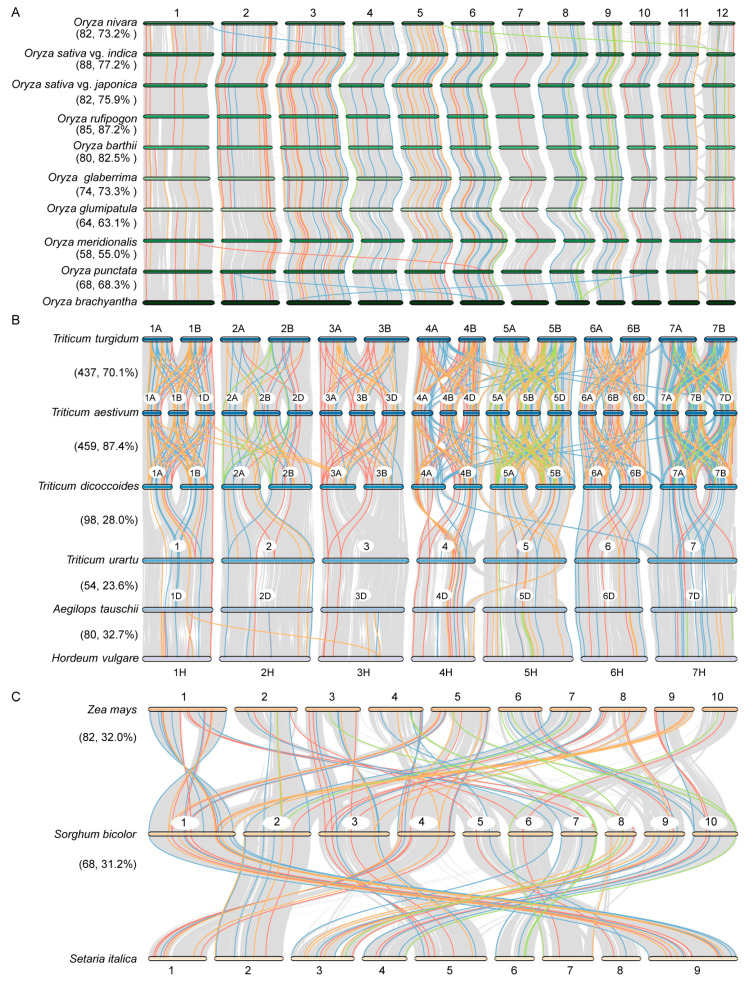
Collinearity analyses of *HSP* genes in *Oryza*, *Triticum*, and *Panicum*. Syntenic analysis of the *HSP* genes in *Oryza* (**A**), *Triticum* (**B**), and *Panicum* (**C**). The collinear genes of *HSP20*, *HSP60*, *HSP70*, and *HSP90* are indicated by the red, blue, orange, and green lines, respectively. The numbers represent the corresponding chromosome numbers. The gray lines indicate the collinearity blocks of genomes between species. The left labels of the figure indicate the species names. The right labels of the figure indicate the number and percentage of collinearity pairs of *HSP* genes in the genome.

Gene duplication events play a significant role in gene and species evolution. To elucidate the evolutionary relationships of *HSP* genes in *Poaceae*, we analyzed the collinearity to explore the synteny of *HSP* genes in *Oryza*, *Triticum*, and *Panicum*. In *Oryza*, 681 *HSP* collinear gene pairs were identified. *HSP* genes were relatively evenly distributed across 10 species, indicating that their functional roles were largely preserved during the evolution (Figure 4A). In *Triticum*, we identified 1128 syntenic gene pairs (Figure 4B). During the evolution progress, a large number of genome duplication events was occurred in *Triticum aestivum*, *Triticum turgidum*, and *Triticum dicoccoides*, leading to an increase in the number of collinear *HSPs* among species. The number of collinear genes of *HSPs* in diploid wheat species was relatively small. In *Panicum*, we identified 150 syntenic gene pairs (Figure 4C). Between maize and sorghum and sorghum and foxtail millet, there were 82 and 68 syntenic gene pairs, respectively, accounting for 32.0% and 31.2% of the average number of *HSP* genes between these species, indicating that the number of *HSP* genes remained relatively conserved during evolution.

Above all, *HSP* genes have experienced complex evolution in *Poaceae*, reflecting the dynamic influence of gene duplication and functional conservation across different species.

### 2.4. Cis-Acting Element Analysis of HSP Genes in Poaceae

**Figure 5:**
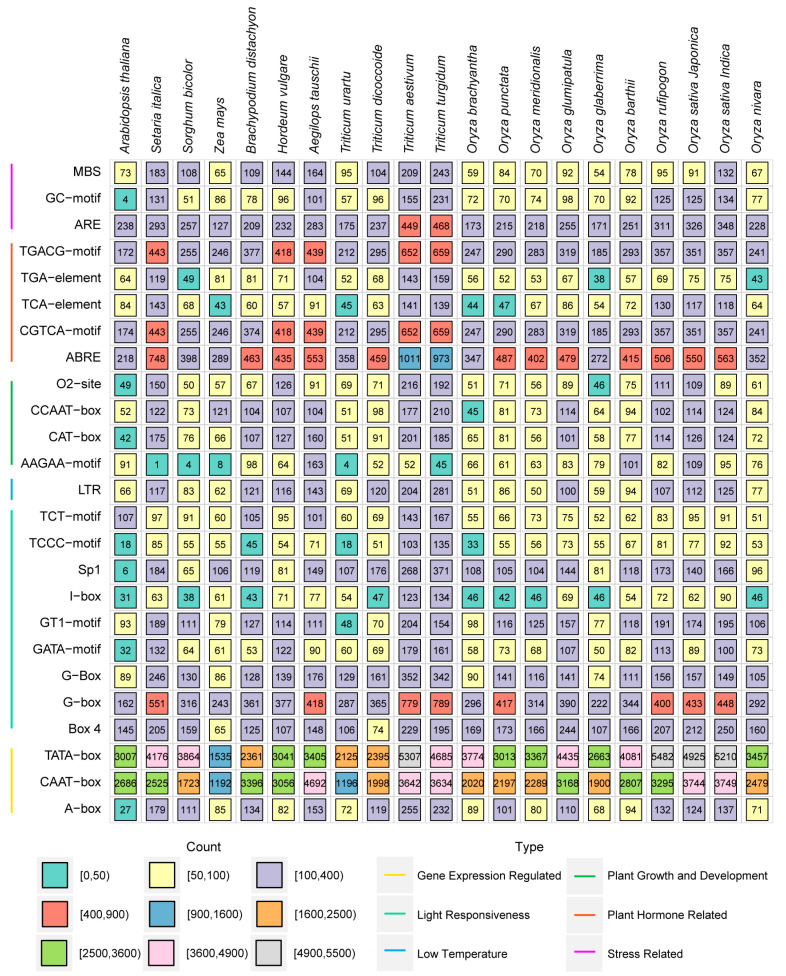
Cis-acting element predicted analysis in *HSP* genes. The cis-acting elements were divided into six groups: gene expression-regulated, light responsiveness, low-temperature, plant growth and development, plant hormone-related, and stress-related. The horizontal axis shows the 21 plant species used in this study, and the vertical axis shows the cis-acting elements of the promoters of *HSP* genes. Different-colored squares indicate the numbers of different cis-acting elements. Different-colored lines indicate cis-acting elements with different functions.

To explore the possible functions and expression regulation of HSP in *Poaceae*, we extracted the 2 kb upstream promoter sequences of *HSP* genes and predicted cis-acting elements using PlantCARE [50] (Figure 5). The top 25 cis-acting elements were further analyzed and were divided to six types, including gene expression regulation, light responsiveness, low temperature-related (LTR), plant growth and development, plant hormone-related, and stress-related (Figure 5). Among these cis-acting elements, gene expression regulation-related elements include the TATA-box, CAAT-box, and A-box. They are essential components of gene promoters and serve as binding sites for RNA polymerase II and transcription factors, playing a pivotal role in regulating gene expression by influencing transcription initiation and the levels of gene expression [53]. Plant hormone-related elements included auxin (TGA-element), salicylic acid (TCA-element), and methyl jasmonate (CGTCA-motif and TGACG-motif) elements (Figure 5). The elements responding to abscisic acid (ABA) stress (ABRE) and methyl jasmonate (MeJA) are abundant in *Triticum*, followed by *Oryza* and *Panicum* (Figure 5). For elements responding to auxin, the distribution in *Oryza* is relatively uniform, while there are differences in the distribution among *Triticum*. Stress response-related elements such as LTR and anaerobic response elements (AREs) were identified in all species (Figure 5).

### 2.5. Functional Conservation Among HSP Members

**Figure 6:**
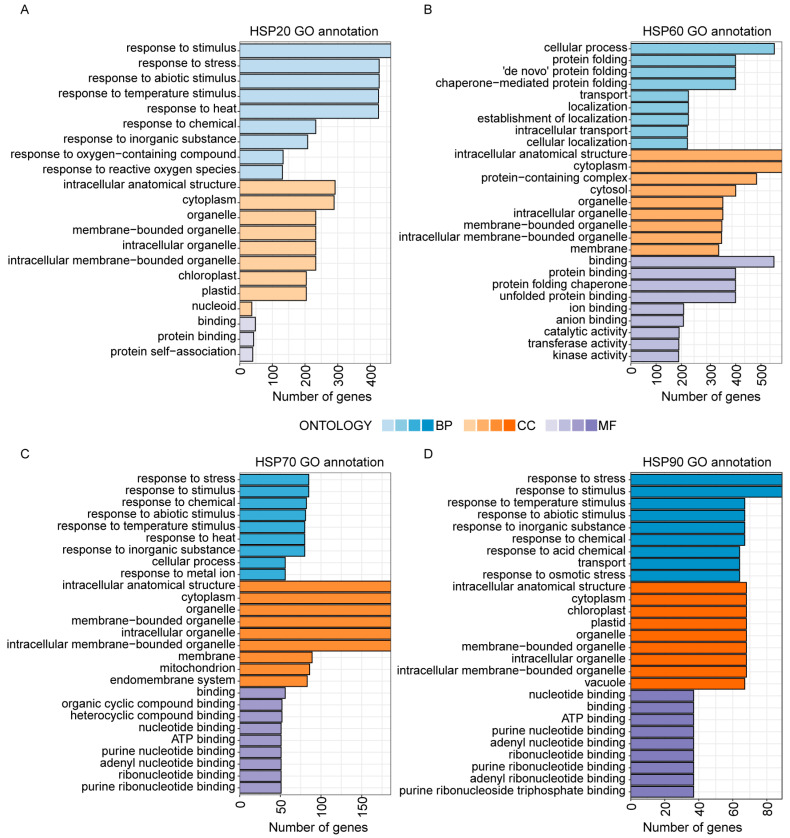
GO annotation analysis of HSPs. (**A**–**D**) show the GO functional annotations of HSP20, HSP60, HSP70, and HSP90, respectively. GO terms were divided into three categories: Biological Process (BP), Cellular Components (CC), and Molecular Function (MF). The vertical axis shows the annotated GO terms. The horizontal axis shows the number of genes corresponding to the GO Terms. Blue, orange, and purple indicate BP, CC, and MF, respectively.

To explore the biological function of HSPs, we performed Gene Ontology (GO) functional annotation of HSPs in *Poaceae* using eggNOG-mapper v2 [54]. We observed that HSP20, HSP70, and HSP90 were primarily enriched in biological process (BP) pathways related to responses to stimulus, stress, and temperature stimulation, indicating that they play roles in stress responses. The cellular components (CC) primarily associated with HSP20, HSP60, HSP70, and HSP90 include cytoplasm, organelles, chloroplasts, and plastids, which corroborate the finding that HSPs are functional in the cytoplasm, chloroplasts, mitochondria, and cell nucleus. The molecular functions (MF) in HSP20, HSP60, HSP70, and HSP90 mainly involve protein binding, which is consistent with their roles as molecular chaperones in regulating protein folding and binding (Figure 6). Further analyses revealed that different subfamilies vary in their distribution across GO functional annotation. For instance, the BP of HSP60 is mainly focused on cellular processes, protein folding, and transport, suggesting biological functions distinct from those of other HSP subfamilies (Figure 6B).

### 2.6. Transcriptomic Analysis of HSP Expression in Maize and Wheat Under Cold Stress Using RNA-Seq

**Figure 7:**
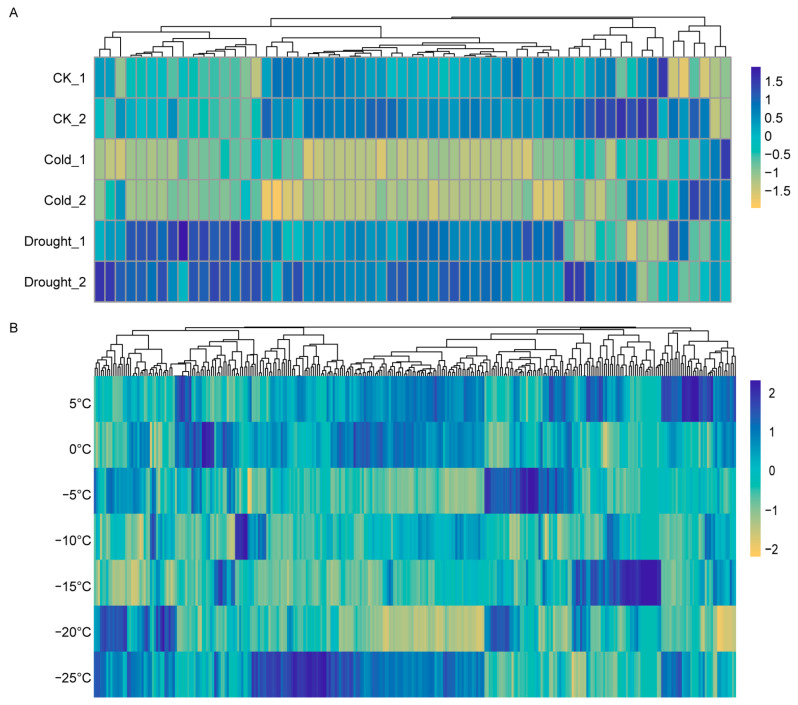
Transcriptional analyses of the collinear *HSPs* in maize and wheat in different treatments. (**A**) Expression profiles of duplicated *ZmHSPs* under cold or drought conditions. The data were extracted from previously published data [55]. (**B**) Expression profiles of duplicated TaHSPs under low-temperature (LT) treatment at 5 °C, 0 °C, −5 °C, −10 °C, −15 °C, −20 °C, and −25 °C. The data were extracted from previously published data [56]. The data are shown in a heatmap with gene expression levels in different treatments with row-scaled FPKM values. The legend is shown at the right of (**A**,**B**).

To investigate the potential biological functions of collinear *HSP* genes, we further analyzed the expression patterns of the collinear *HSP* genes in maize seedlings and wheat tillering nodes under cold stress using previously published transcriptome data [55,56] (Figure 6). In the previous study, thirteen-day-old maize seedlings were treated for 12 h at 4 °C for cold stress treatment or treated with 20% PEG2000 3 h for drought stress treatment. Seedlings grown under the same conditions without any stress treatment were used as the control group (CK). We extracted the expression levels of collinear *ZmHSP* genes from these RNA-seq data and found that these genes displayed differential expression patterns (Figure 7A) [55]. Notably, under low-temperature treatment, the expression levels were markedly downregulated, while under drought treatment, their expression levels were upregulated (Figure 7A). This suggests that the collinear *ZmHSP* genes may exhibit distinct biological functions under different abiotic stresses. We also extracted and analyzed the expression dynamics of the collinear *HSP* genes under serial cold treatments of a winter wheat variety, Dn1 [56]. The plants were treated with low temperatures ranging from 5 °C to −25 °C, and then, the tillering nodes were collected and used for further analysis [56]. We found that the collinear *TaHSP* genes exhibited diverse expression patterns (Figure 7B). More than half of the *TaHSP* genes showed increased diverse expression patterns under the −25 °C treatment condition, indicating that more collinear *TaHSP* genes responded to the −25 °C treatment (Figure 7B).

In summary, transcriptome data from maize and wheat reveal that many collinear *HSP* genes respond to low-temperature treatments.

### 2.7. Expression Patterns of OsHSP Under Cold Stress

**Figure 8:**
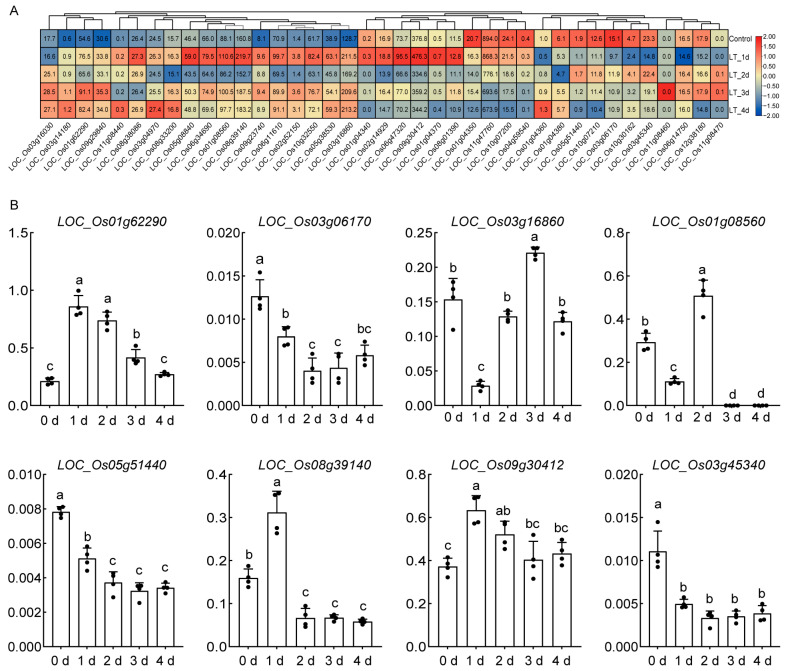
The expression pattern of *OsHSP* genes under cold treatment. (**A**) Expression pattern of collinear *OsHSPs* from RNA-seq data in anther [57] under low-temperature (LT) treatment for 1, 2, 3, and 4 days. The legend is shown at the right of A. (**B**) Expression levels of *OsHSPs* under low temperature. Whole seedlings before (0 day) and after 1 d, 2 d, 3 d, and 4 d of treatment under 17 °C were collected for RT-qPCR analysis. *X*-axis represents the days of cold treatment. The expression levels were calculated using 2^−ΔΔCt^ methods using *OsActin1* (Os03g0718100) as the reference gene. Means +SDs from four biological replicates are represented. Different letters indicate significant differences according to one-way analysis of variance (ANOVA).

To analyze the expression dynamics of the collinear *OsHSP* genes in rice, we extracted their expression levels from previously published RNA-seq data, in which a low-temperature-sensitive rice variety, DN428, was selected [57]. Qu et al. dissected the anthers from a plant treated at 17 °C for 0 (CTL sample), 1, 2, 3, and 4 days and performed an RNA-seq analysis [57]. By analyzing this dataset, we found that among the total 39 collinear *OsHSP* genes, 23 were upregulated after 1 d treatment and 10 showed upregulation after 2 d treatment. After 3 and 4 d of low-temperature treatment, the expression pattern of collinear *OsHSP* genes was similar, which showed consistent upregulation and downregulation, in contrast to the different expression pattern after 1 or 2 d of treatment. For example, *LOC_Os08g39140* was upregulated after 1 day of low-temperature treatment, downregulated on day 2, and upregulated on days 3 and 4 (Figure 8A). These results indicate that these collinear genes display varied response patterns under low-temperature conditions. To gain insight into the expression dynamics of these genes in seedlings at low temperatures, we also measured the expression levels of eight randomly selected collinear genes. We exposed 10-day-old rice seedlings to low temperature (17 °C) for 0 to 4 days. As shown in Figure 8B, the expression levels of *LOC_Os01g62290*, *LOC_Os08g39140,* and *LOC_Os09g30412* were upregulated after 1d of low-temperature treatment, whereas the expression of *LOC_Os03g06170*, *LOC_Os03g16860*, *LOC_Os01g08560*, *LOC_Os05g51440*, and *LOC_Os05g51440* was downregulated after 1d of low-temperature treatment (Figure 8B). Regarding some collinear *OsHSP* genes, the same gene showed varied expression patterns in different cold treatment conditions. This suggests that these *OsHSP* genes may play different biological roles in the cold response.

## 3. Discussion

Heat shock proteins (HSPs) are essential and responsive to various stresses, such as low and high temperatures, osmotic stress, drought, salinity, ultraviolet radiation, and mechanical injury [33]. Although HSP families have been identified in several species, the identification and evolutionary relationships of HSPs in *Poaceae* remain incomplete. In this study, we identified 4730 *HSP* genes across 36 representative species, including algae (34–45), mosses (93), lycophytes (71), basal angiosperms (79), monocots (88–384), and eudicots (79–316). We explored the phylogenetic relationships of HSPs, gene duplication events, cis-elements in promoter sequences, and expression patterns under cold stress in rice, maize, and wheat. Our results revealed that HSPs are widely distributed from aquatic to terrestrial plants, with variations in member numbers during their evolution (Figure 1). Differences in the number of HSP family members were observed among *Poaceae* species, which may be attributed to differences in their phylogenetic patterns and gene duplication events. For HSP20, the phylogenetic tree results showed that the UAP VI subfamily was not identified in wheat, while it was present in rice and millet, suggesting that the UAP VI subfamily may have been lost during evolution in wheat (Figure 2E) [40,58]. The II-like subfamily of HSP70 was uniquely identified in *Poaceae*, indicating its potential emergence during Poaceae evolution [42]. The presence of members of the IV and V-like subfamily of HSP70 in rice and wheat suggests that these two subfamilies may have evolved after the divergence of *Panicum*. Additionally, the loss of the IV and V-like subfamily in *Panicum* may also be attributed to evolutionary processes (Figure 2E) [59]. Previous studies have classified HSP90 into five groups [34]. The results of the phylogenetic relationship in HSP90 showed that no Group 1 subfamily members were identified in rice, suggesting that the Group 1 subfamily was lost during evolution in rice. The number of Group 2 subfamily members in rice was higher than that in wheat and millet, indicating that Group 2 subfamily members expanded during the evolutionary process (Figure 2E). These results suggest that Group 2 subfamily members may play additional biological functions in rice.

Gene duplication provides a basis for functional diversification and enhance plants’ ability to cope with environmental stresses. We analyzed the gene duplication events in the HSP family in *Poaceae*. Most of the *HSP* genes resulted from dispersed duplication, with whole-genome duplication (WGD) and tandem duplication (TD) being less common (Figure 3B). Dispersed segmental duplication and genome polyploidization likely increased the number of *HSP* genes and expanded the HSP family (Figure 3B). Most duplicated *HSP* genes across the species showed Ka/Ks ratios below 1, indicating conserved functions. However, some genes with Ka/Ks ratios above 1 were identified, suggesting positive selection during evolution (Figure 3F). A comparative genomics analysis revealed gradual increases in collinear *HSP* genes in *Oryza*. *Triticeae* showed a significant expansion of collinear *HSP* genes during polyploidization from diploid to polyploid. In contrast, *Panicoideae* maintained relatively stable numbers of collinear *HSP* genes (Figure 4). These results indicates complex *HSP* gene evolution in *Poaceae*.

The promoter cis-acting element and GO function annotations indicated that HSPs are involved in stress responses (Figure 5 and Figure 6). The analysis of cis-acting elements in *HSP* genes involved gene expression regulation and low-temperature and phytohormone responses (Figure 5). Glutathione (GSH) influences the expression of *HSP* genes by regulating the transcription factors MYB21 and bZIP10. GSH also enhances the chaperone function of HSPs by enhancing the interaction between HSP70 and glutathione S-transferase (GST), helping GST maintain its proper conformation and increasing plant tolerance to oxidative stress. The GSH mutant *pad2.1* shows low *HSP* gene expression and is more sensitive to environmental stresses like cadmium and oxidative stress [60]. The results of the GO functional annotation indicated that all HSPs were associated with stress responses, stimulus responses, and protein binding (Figure 6). Previous studies have found that AtHSP90 is not only involved in plant growth and development but also the abiotic stress response. The expression of *HSP90* in *Arabidopsis* increased after undergoing heat, salt, and heavy metal stresses [61]. HSP90 maintains the stability of the R protein RPS2 by binding to it, preventing degradation [62]. After HSP90 activity is inhibited by geldanamycin (GDA), the RPS2-mediated hypersensitive response (HR) and disease resistance are significantly reduced. In *athsp90.1*, RPS2 resistance is defective, and bacterial growth increases [62]. In rice, the *OsHSP90* expression was also induced by salt, drought, low temperatures, and ABA signaling [63].

In recent years, low-temperature stress has emerged as a prevalent abiotic stress in agricultural production. It disrupts plant growth and development and can directly or indirectly impact physiological functions, cell membrane components, and the structure of plants [6,64]. Consequently, this leads to a significant reduction in yield and quality, severely hampering agricultural production. To combat these detrimental impacts, plants modulate the expression of *HSPs*, which are vital in alleviating low-temperature stress [65,66]. Numerous *HSPs* are upregulated in response to low-temperature stress in plants such as *Arabidopsis* [67], rapeseed [68], tobacco [69], maize [70], wheat [71], barley [72], and chicory [73]. Under low-temperature stress, some HSPs are induced and translocated to various cellular compartments to protect cells from damage [67]. In *Arabidopsis*, *HSP70-1* expression was upregulated after 6 h of treatment at 4 °C, with HSP70 relocating from the cytoplasm to the nucleus [67]. In peas, *HSP22* and *HSP70* were significantly increased after 36 h of treatment at 4 °C [74]. In poplar, *HSPs* accumulate in leaves at low temperatures [75]. In rice, the *HSP95* expression in chloroplasts increased with a gradual decrease in temperature from 15 °C to 0 °C, accompanied by the accumulation of HSP75 and HSP70 proteins [76]. In wheat and sunflower, the abundance of HSP90 and HSP60/HSP21 proteins were increased under low-temperature conditions [77]. Low temperatures promote reactive oxygen species (ROS) accumulation, which may damage cells [78]. Cold stress might induce HSPs to stabilize protein structures. HSP17.2 has antioxidant properties. Overexpressing *HSP17.2* in lilies boosts catalase, peroxidase, and superoxide dismutase activity. This enhances ROS scavenging at high temperatures, reducing cell damage from oxidative stress [79].

The transcriptomic analysis of rice, wheat, and maize under low-temperature stress revealed that *HSP* genes with collinearity exhibited different expression patterns. In maize, most collinear genes showed downregulated expression under low-temperature stress but upregulated expression under drought stress, indicating their distinct roles in different abiotic stress responses (Figure 7A). In wheat, *TaHSP* genes were specifically induced under cold (5 °C) and freezing (0 °C, −5 °C, −10 °C, −15 °C, −20 °C, −25 °C) stress, with variable expression patterns across different temperatures (Figure 7B). In rice, the expression of most *HSP* genes under low-temperature treatment was responsive to cold (Figure 8A). Additionally, the expression pattern of collinear *OsHSP* genes according to qRT-PCR (Figure 8B) was not completely equal to that according to RNA-seq data, and the difference in expression pattern may be caused by multiple reasons. This difference likely arises from the distinct varieties used in the two analyses. The RNA-seq data came from DN428, a cold-stress-tolerant rice variety. In contrast, the qRT-PCR analysis used the *Nipponbare* (Nip) variety. Given these differences, the expression patterns of DN428 may not align with those of Nip under cold stress. These results demonstrate that the same *HSP* gene may function differently across varieties (Figure 8). *HSP* genes show varied expression patterns under different cold treatments, indicating they are specifically regulated in low-temperature conditions and play a key role in the cold response. However, the biological functions of these HSPs still require further exploration.

Extreme temperatures can alter developmental growth, physiological mechanisms, metabolism by dysfunctional proteins, which is a major consequence of lower crop productivity. Heat stress significantly impacts many cultivated plants, causing reduced germination, growth inhibition, abnormal seedlings, and weak seedling vigor [80]. High temperatures can also cause visible damage, such as fruit and leaf discoloration, branch and leaf scorching, and premature leaf aging [81]. When temperatures rise above optimal levels, they disrupt biochemical processes, speeding up development and shortening growing seasons, which can lower yields [82]. In maize, temperatures above 37 °C impair embryo protein synthesis, reducing germination rates, while temperatures of 45 °C can halt coleoptile growth entirely [83,84]. HSP70s play roles in protein metabolism during seed germination. After seeds dry out during high temperatures, unfolded or misfolded proteins can clump together when the seed absorbs water. HSP70s help prevent this harmful clumping by acting as chaperones in all cellular compartments after rehydration. HSP70s protect the active synthesis and transport of proteins, ensuring the metabolic processes during germination [85].

In wheat, TaHSP17.4 enhances plant resilience to multiple stresses, including drought, salinity, and heat, by interacting with TaHOP to activate antioxidant systems and ABA signaling pathways. This highlights the critical role of HSPs in regulating cellular redox balance and stress signaling, providing a breeding target for wheat to cope with extreme environmental conditions like drought and soil salinization. Integrating TaHSP17.4 into wheat by transgenic or marker-assisted selection can reduce yield losses under stress [86]. In soybean, GmHSP17.9 plays a role in improving nitrogen fixation by regulating nodule development and symbiosis. As a molecular chaperone, it helps maintain the activity of sucrose synthase GmNOD100, which ensures proper carbon metabolism and energy supply in the nodules. This enhances nitrogenase activity and stabilizes the nodules, allowing soybean plants to use nitrogen more efficiently and rely less on nitrogen fertilizers [87]. This mechanism supports “green nitrogen fixation” in agriculture, reducing pollution from nitrogen fertilizers.

These mechanisms reveal that HSPs function through protein interaction networks, forming functional complexes to regulate metabolic or signaling pathways. This provides a universal strategy for improving stress resilience and yield across crops. For instance, using wheat stress-resistance genes to enhance stress adaptability in multiple crops, could avoid traditional breeding bottlenecks. These results suggest *HSP* genes’ potential value in agricultural production, but their specific functions still require further exploration.

## 4. Materials and Methods

### 4.1. Acquisition of Plant Genomic Data and Genome-Wide Identification of HSPs

Genome, protein, cDNA, and CDS sequences of 36 representative species involved in this study were downloaded from Ensembl Plants, Phytozome13, and WheatGene databases, respectively. The representative plants used in this study were involved in plant taxonomic units including algae, mosses, lithophytes, basal angiosperms, monocotyledons, and dicotyledons. If there was one gene ID corresponding to multiple protein isoforms, the longest isoform was selected using Python 13 to ensure one gene ID corresponded to a single protein. The protein sequences of HSPs in *Arabidopsis thaliana* were obtained from TAIR and OsHSPs were obtained from the Rice Genome Annotation Project (RGAP). The annotated HSPs from Arabidopsis thaliana and rice were BLASTed to the proteomes generated for all the 36 representative species using (https://blast.ncbi.nlm.nih.gov/, accessed on 10 July 2024). The HSPs were identified using the screening criteria (E-value < 1 × 10^−10^ and an identity > 60%).

Hidden Markov models of HSP structural domains were derived from the PFAM database, including the HSP20 structural domain (PF0001), HSP60 structural domain (PF00118), HSP70 structural domain (PF00012), and HSP90 structural domain (PF00183). The hmmsearch program in the HMMER software was used to screen the HSP20, HSP60, HSP70, and HSP90 proteins according to the Hidden Markov models from the proteomes generated for the 36 representative species [88]. The obtained HSP protein sequences were further validated using the NCBI Conserved Domain Database (http://www.ncbi.nlm.nih.gov/Structure/cdd/wrpsb.cgi, accessed on 12 July 2024), PFAM (http://pfam-legacy.xfam.org/, accessed on 13 July 2024), and SMART (http://smart.embl-heidelberg.de/, accessed on 13 July 2024).

### 4.2. Phylogenetic Analysis of HSP Genes

Multiple sequence alignments were performed using the MAFFT software v7.487 [89]. A maximum likelihood tree was constructed using the Randomized Axelerated Maximum Likelihood (RaxML) with the PROTGAMMAJTT amino acid model and a bootstrap value set to 1000. An ML tree was constructed by using RaxML(v1.2.0) with the parameters “-T 40 -n HSP90-m PROTGAMMAJTT-# 1000-x 123-p 123-f a” [90]. The phylogenetic tree was visualized using the R package ggtree v3.2.1.

### 4.3. Chromosomal Distribution, Gene Duplication, Ka/Ks, and Synteny Analysis

Chromosomal distribution information for HSP genes from each species was obtained from the genome annotations. Gene duplication and synteny analyses were performed using the MCScanX 0.8 software with the default parameters [91]. The duplicate_gene_classifier program was used to classify gene duplication types, including singleton (SL), dispersed duplication (DSD), proximal duplication (PD), tandem duplication (TD), and whole-genome duplication (WGD). Synteny analysis and visualization across species were conducted using JCVI 1.4.21 [92]. The protein sequences and CDS sequences of HSP genes with gene duplication were aligned using the ClustalW 2.1 software [93]. The synonymous substitution rate (Ks), nonsynonymous substitution rate (Ka), and evolutionary ratio (Ka/Ks) between HSP gene duplicate pairs were calculated using KaKs_Calculator 2.0 [94].

### 4.4. Analysis of Cis-Acting Element HSP Genes

The PlantCare database (http://bioinformatics.psb.ugent.be/webtools/plantcare/html/, accessed on 15 April 2025) were used for analyzing cis-acting elements [50]. The 2 kb upstream region from the translation start initiation codon, ATG, was used. All results were visualized in R 4.3.0 software.

### 4.5. GO Functional Annotation

The GO functional annotations were conducted using the eggNOG-mapper database (http://eggnog-mapper.embl.de/, accessed on 15 April 2025) [54]. Statistical analysis and visualization were conducted using R 4.3.0.

### 4.6. Expression Pattern Analysis of HSP Genes Using RNA-seq Data

Transcriptome data for rice, maize, and wheat under low-temperature conditions were downloaded from the NCBI-Sequence Read Archive (SRA) database: PRJNA772921 [57], PRJNA309111 [55], and PRJNA787922 [56]. The transcriptome data were aligned and quantified using Hisat2 2.2.0 [95] and featureCount 1.6.4 in Subread (https://subread.sourceforge.net/featureCounts.html, accessed on 15 April 2025). The expression patterns were visualized using the R package pheatmap.

### 4.7. RNA Isolation and Expression Analysis of OsHSP Genes by RT-qPCR

The seeds of the rice (*Oryza sativa* L. ssp. *japonica*) variety *Nipponbare* (Nip) were germinated and grown in a growth chamber at 30 °C under a 16 h light/8 h dark regime for 10 days in Yoshida rice nutrient salt solution (pH 5.5–5.8). The seedlings were transferred into a plant growth chamber (12 h of light/12 h of dark) at 17 °C for 1 day (1 d), 2 days (2 d), 3 days (3 d), and 4 days (4 d) to undergo cold treatment; the seedlings before the transfer were used as the untreated plants (0 day). Total RNA from whole seedlings was extracted by using Trizol (Life Technologies, Carlsbad, California, USA, Product Number 15596-026). Genomic DNA contamination was removed by DNase I (Roche, Basel, Switzerland, Product Number 04716728001) following the manufacturer’s guidelines. Chloroform extraction and ethanol precipitation were performed to purify the RNA after DNase I treatment. A total of 2 μg RNA used to generate cDNA using the HiScript II 1st Strand cDNA Synthesis Kit (Vazyme, Nanjing, China, Product Number R211-01) with Oligo dT (15, 18, and 21). The cDNA was then diluted 10 times for qPCR. The ChamQ SYBR qPCR Master Mix (Vazyme, Nanjing, China, Product Number Q311-02) kit was used. *OsActin1* (Os03g0718100) was used as the reference gene. The expression levels were calculated using 2^−ΔΔCt^ methods from four biological replicates.

## 5. Conclusions

Heat shock proteins (HSPs) play a pivotal role in stress responses. In this study, we employed comparative genomics to identify HSPs across 36 representative plant species, revealing their widespread distribution from aquatic to terrestrial plants. The analysis showed a progressive increase in HSPs during evolution, which is probably driven by dispersed duplication (DSD) and tandem duplication (TD). Notably, as the number of HSPs increased, the protein interaction networks became more complex. We compared the expression levels of the collinear HSPs by reanalyzing the published RNA-seq data. These collinear HSPs exhibited significantly differential expression patterns at low temperatures in rice, maize, and wheat, highlighting their potential for genetic engineering to enhance cold tolerance in crops. This study enhances our understanding of the evolutionary changes in HSPs in *Poaceae* but also provides a valuable resource for future research aimed at improving crop resistance stress conditions.

## Data Availability

The data are contained within the article or Supplementary Materials.

## Supplementary Materials

The following supporting information can be downloaded at: https://www.mdpi.com/article/10.3390/ijms26094269/s1.
