# Supplementary material for: Genome-Wide Identification and Evolution-Profiling Analysis of Heat Shock Protein Gene Family in Poaceae Barnhart
Source: Int J Mol Sci. 2025 Apr 30;26(9):4269. doi: 10.3390/ijms26094269 (PMC12072500; doi:10.3390/ijms26094269)
Supplement: Supplementary file 1 [file ijms-26-04269-s001.zip › Supplemental table S1.pdf]

Supplement Table S1 The detailed information of heat shock proteins (HSPs) member

Type	Cluster	ID
HSP20	CI	TraesCS3A02G033900, TraesCS3A02G034000, TraesCS3D02G045600, TraesCS3A02G034500, TRIDC3AG003760, TraesCS3D02G046700, TraesCS3D02G046800, TraesCS3D02G045500, TRITD3Bv1G014400, TRITD0Uv1G101850, TraesCSU02G175200, TraesCS3D02G045800, TRITD3Bv1G012590, TRITD0Uv1G024000, TraesCSU02G194600, TRIDC3BG006690, TraesCS3B02G049900, TRIDC5BG040910, TRITD5Bv1G141210, TraesCS5B02G245700, TRITD0Uv1G023990, HORVU2Hr1G120170, TraesCSU02G164000, TraesCSU02G047300, AET3Gv20090000, TRIDC3AG005500, AET3Gv20089800, TraesCS3D02G045700, TraesCS3D02G046600, TraesCS3D02G046300, AET3Gv20090900, HORVU3Hr1G007500, TRIDC3BG006670, TraesCS3B02G049800, HORVU3Hr1G006530, HORVU3Hr1G007380, Bradi2g02400, Bradi2g02410, Bradi2g12990, Zm00001d008577, Zm00001d039936, Zm00001d039933, Sobic.003G081900, Sobic.003G082000, Seita.5G093000, Seita.5G092900, Sobic.003G082200, Seita.5G092700, OB01G12240, OB01G12230, OMERI01G02230, OGLUM01G02530, OBART01G02330, LOC_Os01g04370, ORUFI01G02400, ORGLA01G0020000, OPUNC01G02000, OGLUM01G02520, BGIOSGA002444, OPUNC01G02010, LOC_Os01g04380, ONIVA01G02670, ORUFI01G02420, BGIOSGA002723, ORGLA01G0020100, OBART01G02340, OMERI01G02240, ORGLA01G0019900, OBART01G02320, LOC_Os01g04360, ONIVA01G02500, ORUFI01G02390, BGIOSGA002445, OMERI01G02220, OGLUM01G02510, OPUNC01G01990, OB01G12220, Sobic.003G082100, Seita.5G092800, TRIDC3AG003790, TRITD3Av1G008010, TraesCS3A02G035400, AET3Gv20088300, TraesCS3B02G048800, TRIDC3BG006550, TRITD3Bv1G012460, TRIDC3AG003800, TraesCS3A02G035500, TuG1812G0300000397, TraesCS3B02G048700, TRIDC3BG006530, TRITD3Bv1G012440, AET3Gv20088200, HORVU3Hr1G006930, HORVU3Hr1G006940, Bradi2g02350, Sobic.003G082300, Zm00001d039941, Seita.5G092600, ORGLA01G0019800, OBART01G02310, OGLUM01G02490, BGIOSGA002722, OPUNC01G01980, OB01G12210, LOC_Os01g04350, OMERI05G14900, ORUFI01G02380, OMERI01G02210, ONIVA01G02490, OBART01G02300, ONIVA01G02480, LOC_Os01g04340, ORUFI01G02370, ORGLA01G0019700, BGIOSGA002721, OMERI01G02200, OGLUM01G02480, OPUNC01G01970, OB01G12200, TRITD0Uv1G106830, TRITD3Bv1G012430, TRIDC3BG006470, TraesCS3B02G048600, TraesCS3D02G044400, AET3Gv20087400, Sobic.003G082500, Seita.5G092400, Zm00001d039942, AT2G19310, AT1G53540, AT3G46230, AT5G59720, AT1G59860, AT1G07400, AT2G29500, TraesCS4A02G092700, TRIDC4AG012590, TRITD4Av1G041570, TRIDC4AG012580, TraesCS4A02G092600, TraesCS4D02G212500, AET4Gv20541200, TRIDC4BG037720, HORVU4Hr1G060760, Bradi1g67040, Seita.9G458700, Zm00001d028561, Sobic.001G425500, Zm00001d028557, Zm00001d028555, Sobic.001G426000, Seita.9G458200, Sobic.001G425600, Zm00001d047841, Seita.9G458600, TRITD4Av1G041810

Type	Cluster	ID
HSP20	CI	TRIDC4AG012630, TraesCS4A02G092900, TuG1812G0400002579, AET4Gv20540900, TraesCS4D02G212200, TraesCS4B02G211600, TRITD4Bv1G129390, TraesCS4A02G092800, TRIDC4AG012610, TraesCS4D02G212400, TraesCS4D02G213100, TraesCS4D02G213300, TraesCS4A02G092200, TuG1812G0400002505, TRIDC4AG012520, TuG1812G0400002504, TraesCS4A02G092100, HORVU4Hr1G060720, TRITD4Av1G041210, TRIDC4BG037820, TraesCS4B02G212200, TRITD4Bv1G129640, AET4Gv20543300, AET4Gv20542600, TraesCS4D02G212300, TRIDC4BG037840, TraesCS4B02G212300, Bradi1g53850, Bradi1g67080, LOC_Os03g15960, BGIOSGA012293, ONIVA03G12960, OGLUM07G05310, ORGLA03G0113600, OBART03G11750, OMERI03G11040, ORUFI03G12100, OPUNC03G11310, OGLUM03G11760, OBART03G11810, BGIOSGA012296, LOC_Os03g16030, OMERI03G11090, OPUNC03G11360, ONIVA03G13010, OB03G21950, OMERI03G11080, LOC_Os03g16020, BGIOSGA012297, ORUFI03G12140, OBART03G11800, BGIOSGA011047, BGIOSGA011045, OGLUM03G11750, BGIOSGA011044, ONIVA03G13030, ORUFI03G12160, LOC_Os03g16040, OBART03G11830, ORGLA03G0114100, OMERI03G11100, OPUNC03G11380, TraesCS4B02G211700, TRIDC5BG059960, OB03G21920, OPUNC03G11350
HSP20	CII	BGIOSGA006868, LOC_Os02g12610, ONIVA11G03090, OBART02G08830, ORUFI02G08860, OGLUM02G09000, OMERI02G10100, OPUNC02G07850, OB02G17870, Sobic.004G092700, Sobic.004G092800, Seita.1G032700, TraesCS6D02G171600, AET6Gv20460500, AT5G12020, AT5G12030, OB01G15410, OBART01G05500, ONIVA01G06210, BGIOSGA002242, ORGLA01G0051000, LOC_Os01g08860, ORUFI01G05890, OGLUM01G05970, OPUNC01G05330, Zm00001d047553, Zm00001d047548, Zm00001d047542, Zm00001d008841, Sobic.003G039400, Seita.5G131400, Seita.5G131300, Zm00001d039566, Bradi2g05374, TraesCS4D02G145600, AET4Gv20312400, TRIDC3AG013970, TRITD3Av1G035590, TraesCS3A02G113000, TRIDC7BG055420, TraesCS7B02G347100, TRITD7Bv1G188630, TraesCS3D02G114900, AET3Gv20248600, TRIDC3BG018360, TRITD3Bv1G044350, TraesCS3B02G131200, TraesCS3D02G114700, AET3Gv20248400, TraesCS3D02G114800, TraesCS3B02G131300, TRIDC3BG018370, TRITD3Bv1G044370, TuG1812G0300001272, TRIDC3AG013950, TraesCS3A02G112900, TRITD3Av1G035580, HORVU3Hr1G020390, TRITD3Bv1G044280, TraesCS3B02G130300, TRIDC3BG018290, AET3Gv20248800, TraesCS3D02G115000, TraesCS3A02G113100, TRIDC3AG013980, TraesCS3D02G115300, AET3Gv20250200, TraesCS3D02G115400, AET3Gv20250300, TraesCS3B02G130400, TraesCS4D02G145500, AET4Gv20312300, TRITD3Bv1G044290, HORVU3Hr1G020520, TRITD3Bv1G044330, HORVU3Hr1G020490, AET3Gv20249300, TraesCS3D02G115200, TraesCS3D02G115100, TraesCS3B02G131100, TRITD3Bv1G044340, TRIDC3BG018340, TraesCS3B02G130900, TRIDC3BG018310, TraesCS3B02G131000, TRITD3Bv1G044320, TRIDC3BG018320, HORVU3Hr1G020500, TRIDC3BG018300, TraesCS3B02G130500

Type	Cluster	ID
HSP20	CIII	ORGLA02G0302500, OBART02G34970, OGLUM02G36080, OMERI02G32950, LOC_Os02g54140, ORUFI02G36420, ONIVA02G37040, BGIOSGA009154, OBART01G09900, OPUNC02G32250, OB02G41760, Zm00001d004599, Zm00001d020390, Zm00001d037633, AT1G54050, Zm00001d018298, Sobic.004G321000, Seita.1G342400, TRIDC6AG051210, TRITD6Av1G204330, TuG1812G0600003600, TRIDC6BG059990, TraesCS6B02G374100, TRITD6Bv1G199700, AET6Gv20844400, TraesCS6D02G322300, HORVU6Hr1G082360, TRITD6Av1G204340, AET6Gv20844600, TraesCS6B02G374200, Bradi3g60100, Zm00001d042842
HSP20	ER	TRIDC2AG045550, TraesCS2A02G312900, TRITD2Av1G191220, AET2Gv20711600, TraesCS2D02G311400, HORVU2Hr1G077710, TRIDC2BG048270, TraesCS2B02G329900, TRITD2Bv1G157230, Bradi5g11110, BGIOSGA016481, LOC_Os04g36750, ORUFI04G15230, ONIVA04G12160, OGLUM04G13740, OBART04G13960, OMERI04G12570, OPUNC04G11820, Seita.7G111700, Sobic.006G093500, Zm00001d025508, Zm00001d003554, AT4G10250, Bradi4g21070, LOC_Os11g13980, ORUFI11G08350, ORGLA11G0071200, OBART11G08060, OGLUM11G07890, BGIOSGA034220, ONIVA11G08260, OMERI11G06940, Zm00001d007271, Sobic.005G086400, Seita.8G079900, HORVU4Hr1G015170, TraesCS4D02G086200, AET4Gv20184000, TraesCS4A02G226700, TRITD4Av1G168210, TuG1812G0400000865, TRIDC4AG036000, TRIDC4BG013980, TraesCS4B02G089800, TRITD4Bv1G031570
HSP20	MT1	OBART02G07710,BGIOSGA006925,OGLUM02G07770,ORUFI02G07820,LOC_Os02g10710,ONIVA02G09220,BGIOSGA006929,ORGLA02G0072500,OPUNC02G06920,OB02G17000, Zm00001d015777, Sobic.004G083600,Seita.1G045200,TRITD6Av1G082720,TRIDC6AG026340,TraesCS6A02G181700,TuG1812G0600001860,TRIDC6BG032410,TRITD6Bv1G086480,TraesCS6B02G210600,AET6Gv20455500,TraesCS6D02G169100,Bradi3g07421,AT1G52560,TRITD7Av1G084760,TraesCS7A02G232500,TuG1812G0700002484,TRIDC7AG029910,TraesCS7D02G232600, AET7Gv20574900, TRIDC7BG020650, AET7Gv20460900, TraesCS7D02G179400, TraesCS7B02G082900, TRIDC7BG012360, TraesCS7B02G083400, TRITD7Bv1G033690, TraesCS7B02G083300, TRITD7Bv1G033530, TRIDC7BG012370, HORVU7Hr1G036570, HORVU7Hr1G036500, HORVU7Hr1G036470, HORVU7Hr1G036540,TRITD7Av1G087130, TraesCS7A02G236700, TuG1812G0700002511, TraesCS7D02G179300, AET7Gv20460700, TRITD7Av1G056880, TRIDC7AG022060, TRIDC7AG022040, TuG1812G0700001913, TRITD7Av1G056730, TRITD7Av1G056670, TraesCS7A02G177500, TraesCS7A02G177700, TRITD0Uv1G122340, TRITD7Av1G056890, TuG1812G0700001916, TraesCS7A02G177600, TuG1812G0700001915, TraesCS7D02G179200, TraesCS7D02G179500, TRIDC2BG076470, TraesCS2B02G531300, TRITD2Bv1G235410, TRIDC7AG022050, TRIDC7BG012420, TRIDC7BG012430, TraesCS7B02G083200, TRITD7Bv1G033640, TraesCS7B02G083100, TraesCS7D02G179000, TraesCS7D02G179100, TRIDC7AG022070, TRITD7Av1G056990, TuG1812G0700001920, TRITD7Bv1G033710

Type	Cluster	ID
HSP20	MT1	TraesCS7B02G083500, TRIDC7BG012440, TraesCS7D02G179600, AET7Gv20461000, TraesCS7D02G178900, ONIVA06G08800, ORUFI06G07770, BGIOSGA021653, OGLUM06G08080, LOC_Os06g11610, ORGLA06G0071500, OBART06G07610, OMERI06G08790, OPUNC06G07250, OB06G17180, Sobic.010G057633, Sobic.K000500, Sobic.010G087700, Sobic.010G087800, Zm00001d044874, Sobic.010G087600, Seita.4G089700, Seita.4G079800, Sobic.010G087500, Seita.4G089400, BGIOSGA009084, ORUFI02G34770, OGLUM02G33730, ONIVA02G35740, OBART02G33220, ORGLA02G0287000, LOC_Os02g52150, OMERI02G31700, OPUNC02G30530, OB02G40120, Seita.1G326600, Zm00001d052194, Sobic.004G228900, Bradi3g58590, TRIDC6AG047510, TRITD6Av1G193530, TraesCS6A02G316200, TuG1812G0600003397, AET6Gv20780800, TraesCS6D02G295500, TRITD6Bv1G185560, TRIDC6BG055490, TraesCS6B02G346700, HORVU6Hr1G077710, AT5G51440, AT4G25200
HSP20	P	TRITD4Bv1G136930, TraesCS4B02G225400, TRIDC4BG039790, TraesCS4D02G226000, AET4Gv20569400, TRIDC4AG010780, TraesCS4A02G068300, TRITD4Av1G033110, TuG1812G0400002666, TRIDC4AG010790, TraesCS4A02G068200, TRITD4Av1G033200, TuG1812G0400002668, TuG1812G0400002671, TuG1812G0400002665, TRITD4Av1G033120, HORVU4Hr1G063350, AET4Gv20569700, Bradi1g68440, Zm00001d028408, Sobic.001G438000, Seita.9G470600, LOC_Os03g61940, BGIOSGA009495, ORUFI03G41850, OGLUM03G39920, OBART03G40040, OMERI03G36890, OGLUM03G10130, BGIOSGA012224, ONIVA03G10990, OBART03G10370, LOC_Os03g14180, ORUFI03G10580, OB03G20550, OMERI03G09610, OPUNC03G10020, AT4G27670, ORUFI05G11240, LOC_Os05g23140, OBART08G09200, OGLUM05G10810, ONIVA06G21850, BGIOSGA018362, OMERI05G08870, OPUNC05G06180, OB05G19210, Sobic.007G217300, Zm00001d031325, Seita.6G122600, TraesCS5B02G257000, TRITD5Bv1G145910, TRIDC5BG042700, AET5Gv20605700, TraesCS5D02G266000, TuG1812G0500002945, TraesCS5A02G257700, TRIDC5AG040350, TRITD5Av1G157030
HSP20	P-Like	TraesCS5B02G205300,TRITD5Bv1G125490,TRIDC5BG035220,TraesCS5D02G213100,AET5Gv20505800,TRITD0Uv1G046220,TraesCS5A02G206900,TRIDC5AG033320,TuG1812G0500002333,Bradi3g20830,ORUFI10G02990,LOC_Os10g07210,ONIVA10G03090,OBART10G03890,OMERI10G02530,OMERI03G33630,OGLUM10G03020,OPUNC10G02400,BGIOSGA032265,Zm00001d030346,Sobic.001G264300,Seita.9G285000,Sobic.001G265800,Zm00001d030317,Seita.9G287100,OGLUM09G07130,Sobic.005G010300,LOC_Os10g07200,ORUFI10G02980,ONIVA10G03080,BGIOSGA032268,OBART10G03880,OGLUM10G03030,OPUNC10G02390,BGIOSGA032267,OMERI10G02520,OMERI03G33620,OB10G12200,TRITD5Bv1G125480,TraesCS5B02G205200, TraesCS5D02G213000, AET5Gv20505700, TRITD0Uv1G046230, TraesCS5A02G206800, TRIDC5AG033300, TuG1812G0500002334, HORVU5Hr1G061160, Bradi3g20840, Sobic.001G264400, Zm00001d030345, Seita.9G285100

Type	Cluster	ID
HSP20	PX	ORUFI06G09980, LOC_Os06g14240, BGIOSGA021524, ONIVA06G11150, ORGLA06G0090800, OBART06G09630, OGLUM06G10430, OPUNC06G09210, OB06G18890, TRITD7Bv1G044980, TraesCS7B02G109100, TRIDC7BG015960, TraesCS7A02G202200, TRIDC7AG025560, TRITD7Av1G069250, HORVU0Hr1G020420, TraesCS7D02G205400, AET7Gv20513900, Bradi1g44230, Zm00001d044728, Sobic.010G104300, Seita.4G111400, AT5G37670
HSP20	UAP I	OGLUM07G16020, BGIOSGA025811, ONIVA07G14540, ORUFI07G16780, OBART07G16100, LOC_Os07g33350, ORGLA07G0132600, OMERI07G13160, OPUNC07G15050, Sobic.002G319100, Zm00001d021634, Seita.2G332600, Bradi1g26190, AET2Gv20472400, TraesCS2D02G227800, HORVU2Hr1G046370, TraesCS2B02G247300, TRITD2Bv1G093300, TRIDC2BG033290, AT4G21870
HSP20	UAP II	TraesCS7D02G185500, TraesCS7A02G183800, TraesCS7D02G185600, TRITD7Bv1G037370, TRIDC7BG013260, TraesCS7B02G088600, TraesCS7B02G088700, ORUFI02G31520, ORGLA02G0254300, OBART02G29900, LOC_Os02g48140, BGIOSGA008910, ONIVA02G32660, OMERI02G28740, OPUNC02G27470, OB02G37080, Zm00001d017813, Sobic.004G263100, Seita.1G293800, OBART02G02230, ORGLA02G0021300, BGIOSGA007446, LOC_Os02g03570, ORUFI02G02240, ONIVA02G02150, OGLUM02G02090, OPUNC02G01670, OB02G12140, Zm00001d053965, Sobic.004G026300, Seita.1G101200, TRITD6Av1G022960, TuG1812G0600000888, TRIDC6AG011070, TuG1812G0600000904, TraesCS6A02G087600, TraesCS6B02G110200, TRITD6Bv1G032050, TRIDC6BG015810, HORVU6Hr1G015130, TraesCSU02G019000, AET6Gv20217000, Bradi3g02710
HSP20	UAP IV	OBART05G21820, ORGLA05G0190000, ORUFI05G23080, ONIVA05G22500, LOC_Os05g42120, BGIOSGA020122, OGLUM05G23010, OMERI05G19690, OPUNC05G19410, Zm00001d038608, Sobic.009G187400, Zm00001d010693, Seita.3G189400, TRIDC1AG047590, TraesCS1A02G319600, TuG1812G0100003624, TRIDC1BG053830, TRITD1Bv1G178260, TraesCS1B02G331900, TraesCS1D02G319400, HORVU1Hr1G066530, Bradi2g20767, AT5G54660, AT2G03020, AT4G16540, AT5G47590, AT4G16550, AT4G16560, Bradi1g55550, OPUNC06G19340, AT2G17410
HSP20	UAP V	TraesCS7A02G486100,TRITD7Av1G254690,TraesCS7A02G493600, AET7Gv21186500,TraesCS7D02G472700,TraesCS7B02G389800, TRITD0Uv1G064540,TraesCS7B02G389600, TRIDC7BG061640, TraesCS7D02G472900,AET1Gv21028700, TraesCS1D02G445600, TraesCS1B02G473700,TuG1812G0100004813,TraesCSU02G179200, TraesCSU02G179100,HORVU1Hr1G094510,HORVU2Hr1G038170, TRITD1Av1G227810,TraesCS1A02G437300,TuG1812G0100004788, TraesCS1D02G445000,AET1Gv21027400,TraesCS1B02G471800,TRITD1Bv1G225690,HORVU1Hr1G094440,AET1Gv21027500,TraesCS1D02G445100,TRITD1Bv1G225700,TraesCS1B02G471900,TuG1812G0100004789,HORVU1Hr1G094450,Bradi3g29220, Zm00001d039169, Sobic.009G255200, Seita.3G122700

Type	Cluster	ID
HSP20	UAP V	LOC_Os05g51440, ORUFI05G29880, ONIVA05G29800, ORGLA05G0242800, OGLUM05G29380, OBART05G27910, BGIOSGA020479, OMERI05G23810, OPUNC05G25510, OB05G35410, TraesCS3A02G210500, TRITD3Av1G134980, TRIDC3AG031370, TuG1812G0300002471, TraesCS3D02G213300, AET3Gv20521700, HORVU3Hr1G051000, Bradi2g42130, Sobic.003G201400, Zm00001d011241, Seita.5G254800, BGIOSGA001318, ONIVA01G23650, LOC_Os01g40550, ORUFI01G23760, OBART01G21040, OGLUM01G24700, OMERI01G19200, OPUNC01G21100, ONIVA01G23620, ORUFI01G23730, OGLUM01G24690, OMERI01G19180, OPUNC01G21090, ORUFI01G23740, LOC_Os01g40530, ONIVA01G23630, BGIOSGA001319, OB01G30600, Sobic.003G201200, Seita.5G254900, Sobic.003G201300, Bradi3g14550, AT1G76770, AT3G10680
HSP20	UAP VI	BGIOSGA010122, ONIVA03G29070, LOC_Os03g45330, ORUFI03G29230, OGLUM03G28730, ORGLA03G0259700, OBART03G28100, OMERI03G24730, OPUNC03G25600, Sobic.001G148800, Seita.9G151100, AT1G54400, AT5G20970, AT2G27140, AT5G04890, AT1G06460
HSP20	UAP VII	BGIOSGA010121, LOC_Os03g45340, OBART03G28110, ORGLA03G0259800, ORUFI03G29240, OGLUM03G28740, OMERI03G24740, ONIVA03G29080, OPUNC03G25610, OB03G35750, Sobic.001G148600, Zm00001d033478, Zm00001d013641, Seita.9G150800, TRITD4Bv1G003750, TraesCS4B02G014900, TRIDC4BG002540, TraesCS4D02G013100, AET4Gv20022700, TRIDC4AG045830, TraesCS4A02G298600, HORVU4Hr1G002290, Bradi1g13261, Seita.9G151000, ONIVA12G18210, OGLUM12G21020, BGIOSGA035802, OPUNC12G17500, OB12G26150, TRITD5Bv1G007930, TraesCS5B02G022700, HORVU5Hr1G006670, HORVU5Hr1G006570, Bradi4g01190, Zm00001d031129, Sobic.008G178901, Seita.3G035400, Seita.3G396200, AET4Gv20668600, TraesCS4D02G272100, TuG1812G0400003164, TRITD4Av1G010720,TRITD4Bv1G162570,TraesCS4B02G273000,TraesCS4B02G273100,HORVU4Hr1G072700,HORVU4Hr1G072680, HORVU4Hr1G072770,TRITD4Av1G010710,TuG1812G0400003140, TraesCS4B02G273200,Bradi1g74620,OBART03G04290,ORGLA03G0040900, LOC_Os03g06170, OGLUM03G04160,ONIVA03G04070, ORUFI03G03980, BGIOSGA011420, OB03G14380, Seita.9G534800, ORGLA10G0098500, OBART10G11190, LOC_Os10g30162, BGIOSGA033012, OGLUM10G10910, OMERI04G07080, OPUNC10G09430, Zm00001d032893, Sobic.001G239400, Seita.9G243500, Bradi3g27262, Bradi3g27350, TRITD1Bv1G086050, TuG1812G0100001607, BGIOSGA033013, ORGLA10G0098700, OBART10G11200, LOC_Os10g30180, ONIVA10G10340, ORUFI10G11690, OGLUM10G10930, OMERI04G07100, OPUNC10G09560, OB10G18530, Sobic.001G239200, Zm00001d014146, Seita.9G243400, TRITD0Uv1G062310, TraesCS7A02G553500, TuG1812G0700005893, TRIDC7AG076900, HORVU1Hr1G035950, AET1Gv20357600, TraesCS1D02G138300, TRITD1Bv1G086060, TraesCS1B02G155900, TRIDC1BG025350, Bradi3g27360

Type	Cluster	ID
HSP60	Class I	HORVU4Hr1G076680, HORVU5Hr1G074160, HORVU2Hr1G107410, TRIDC6AG000770, TuG1812G0600000079, TraesCS6A02G006400, HORVU6Hr1G000180, TraesCS6B02G012100, TRITD6Bv1G002110, TRIDC6BG001350, TraesCS6B02G012000, TRITD6Bv1G001980, TraesCS6B02G011800, TRITD6Av1G001190, AET6Gv20015200, TraesCS6D02G009100, Bradi3g00480, TRITD6Av1G005340, TraesCS6A02G028100, TuG1812G0600000264, AET6Gv20064600, TraesCS6D02G031400, TRIDC6BG004870, HORVU4Hr1G066820, HORVU6Hr1G005220, TRITD6Bv1G007360,TraesCS6B02G039300, BGIOSGA007332, LOC_Os02g01280, ONIVA02G00290, OBART02G00290, OGLUM02G00290, ORGLA02G0002200, OB02G10300, ORUFI02G00310, OMERI02G09310, OPUNC11G05510, Sobic.004G002200, Zm00001d054089, Seita.1G002500, LOC_Os06g02380, BGIOSGA022066, ORUFI06G00900, OGLUM06G00880, OBART06G00910, ORGLA06G0008300, ONIVA06G01270, OMERI06G00880, OPUNC06G00860, OB06G11010, Zm00001d053279, Zm00001d046709, Zm00001d037526, Zm00001d016540, Zm00001d017567, Sobic.010G011200, Zm00001d035937, Zm00001d045544,Seita.4G010900,TraesCS7A02G036200,TRITD7Av1G008550,TuG1812G0700000402, TRIDC7AG003550, TraesCS7D02G032800, AET7Gv20079800, TraesCS4A02G452700, TRITD4Av1G254470, TRIDC4AG066950, HORVU7Hr1G006490, Bradi1g50270, AT3G13470, AT1G55490, AT5G56500, ChrSy.fgenesh.mRNA, ORUFI02G12680, BGIOSGA034997, OBART04G13840, ORGLA04G0108800, OMERI04G12440, ONIVA04G12050, OGLUM04G13600, OPUNC04G11710, OB04G21060, Sobic.001G437801, Zm00001d028409, Seita.7G110500, TuG1812S0000111500, TRIDC2AG045400, TraesCS2A02G311700, TRITD2Av1G190550, TraesCS2D02G310000, AET2Gv20708400, TraesCS2B02G328500, TRITD2Bv1G156220, TRIDC2BG048020, HORVU2Hr1G077410, Bradi5g11240, AT1G26230, OMERI12G05980, OBART12G08490, ORGLA12G0079300, BGIOSGA037230, ONIVA12G08110, LOC_Os12g17910, OGLUM12G09730, ORUFI12G09360, OPUNC12G07720, OB04G12980, Seita.3G283900, Zm00001d031503, Sobic.009G098100, Zm00001d051252, TRIDC2AG005350, TraesCS2D02G049500, TuG1812G0200000477, TRITD0Uv1G010190, TRIDC2BG005840, TRITD2Bv1G014390, AET2Gv20083000, TraesCSU02G009200, HORVU2Hr1G011340, HORVU2Hr1G109400, HORVU2Hr1G007840, Bradi5g02890, AT2G28000, ONIVA01G48070, BGIOSGA009394, OMERI03G38420, OGLUM03G41600, LOC_Os03g64210, ORGLA03G0386900, OBART03G41760, ORUFI03G43550, OPUNC03G38820, OB03G48790, TRITD5Bv1G252020, TraesCS5B02G563900, TRIDC5BG083320, HORVU5Hr1G125130, TraesCS5D02G550700, AET5Gv21215400, TRITD4Av1G207380, TRIDC4AG047670, TraesCS4A02G315500, Bradi1g00730, Zm00001d034919, Sobic.001G003100, Seita.9G002600, TraesCS5B02G368900, TRITD5Bv1G189480, TRIDC5BG057700, AET5Gv20856400, TraesCS5D02G376000, TRITD5Av1G196200, TRIDC5AG053640, TraesCS5A02G366800, HORVU0Hr1G038830, Bradi4g38090, BGIOSGA031277, ONIVA03G25830, OGLUM09G20420, ORGLA09G0159400, OBART09G19740

Type	Cluster	ID
HSP60	Class I	LOC_Os09g38980, ORUFI09G21300, OPUNC10G13700, OPUNC09G18110, OB09G26350, Zm00001d005373, Sobic.002G131000, Seita.2G129300, Zm00001d031414, AT5G18820, AET4Gv20738200, TRIDC4AG055880, TraesCS4A02G409100, TuG1812S0001755700, TuG1812G0400003401, TRITD4Av1G228440, TraesCS4D02G305900, TRITD4Bv1G177600, TRIDC4BG052460, TraesCS4B02G307700, HORVU4Hr1G078820, Bradi1g75520, TraesCS4B02G266200, TRIDC4BG046190, HORVU4Hr1G071510, TuG1812G0400003084, Seita.9G543300, Sobic.001G507500, BGIOSGA011475, LOC_Os03g04970, OBART03G03380,ORUFI03G03120,ONIVA03G03120,ORGLA03G0031500,OGLUM03G03280,OMERI03G03350,OPUNC03G03120,OB03G13410,TraesCS1B02G162300,TRITD1Bv1G091420, TRIDC1BG026450, TraesCS1D02G144000, AET1Gv20372400, TuG1812G0100001970, TRIDC1AG022060, TraesCS1A02G145000, TRITD1Av1G089270,HORVU1Hr1G038680, Bradi3g28070, Zm00001d032789, Seita.9G231400, Zm00001d014090, Sobic.001G228200,OB10G19690,ORUFI10G13120,LOC_Os10g32550, OGLUM10G12210, OMERI10G09390, ONIVA10G11680, BGIOSGA033085,OBART10G12420,ORGLA10G0110100,OPUNC10G10670,AT2G33210,AT3G23990,TuG1812G0100004020,TRITD1Av1G204730,TraesCS1A02G361400, TRIDC1AG053160, TRIDC1BG060810, TraesCS1B02G378000, TRITD1Bv1G196100, AET1Gv20871200, TraesCS1D02G365800, HORVU1Hr1G079910, Bradi2g18260, LOC_Os05g46290, BGIOSGA017649, OGLUM05G25670, ORGLA05G0207300, OB05G31770, Zm00001d038857, Sobic.009G214100, Seita.3G163700, AT3G13860, Zm00001d042608
HSP60	Class II	TraesCS2B02G217700, TRITD2Bv1G076930, TRIDC2BG028870, TraesCS2D02G198300, AET2Gv20399100, TRITD2Av1G066460, TRIDC2AG024290, TraesCS2A02G189500, TuG1812G0200002000, HORVU2Hr1G036380, Bradi3g36397, LOC_Os08g33200, ORUFI08G17350, BGIOSGA026950, ONIVA08G16540, ORGLA08G0134900, OBART08G15420, OGLUM08G16180, OMERI08G12690, OPUNC08G13480, OB08G22930, Sobic.007G133000, Zm00001d032238, Seita.6G154600, ONIVA09G09280, BGIOSGA029792, ORUFI09G10420, LOC_Os09g23740, ORGLA09G0071800, OBART09G09740, OGLUM09G09970, OMERI09G07520, OPUNC09G08200, TRIDC5AG033000, TraesCS5A02G204600, TRITD0Uv1G039230, TuG1812G0500002380, TraesCS5D02G211500, AET5Gv20501800, TraesCS5B02G203700, TRIDC5BG034940, TRITD5Bv1G124660, Sobic.002G196600, Zm00001d020414, Seita.2G200700, OBART06G09950, ORGLA06G0093900, ORUFI06G10360, ONIVA06G11520, OGLUM06G10810, LOC_Os06g14750, BGIOSGA021504, OMERI06G11630, OPUNC06G09530, OB06G19150, Zm00001d037332,Sobic.010G107000,Seita.4G115600, TraesCS7A02G205300,TRIDC7AG025870,TuG1812G0700002189,TRITD7Av1G070520,TraesCS7D02G208200, AET7Gv20519500, TraesCS7B02G112700, TRIDC7BG016470, TRITD7Bv1G046650, HORVU7Hr1G041920, Bradi1g44047, AT1G71010, TRITD7Av1G145520, TRIDC7AG040560, TraesCS7A02G294300, TuG1812G0700002974, AET7Gv20728300, TraesCS7D02G289700, TRIDC7BG030040, TraesCS7B02G184000, TRITD7Bv1G092420

Type	Cluster	ID
HSP60	Class II	HORVU7Hr1G069660, Bradi3g37350, ORGLA09G0169600, ORGLA08G0145800, OBART08G16730, ORUFI08G18590, LOC_Os08g34950, ONIVA08G18250, BGIOSGA028768, OGLUM08G17460, OMERI01G15590, OPUNC08G14630, OB08G24130, Zm00001d050089,Sobic.007G146400,Seita.6G168700, TRITD4Bv1G089770, TraesCS4B02G152100, TRIDC4BG027850, AET4Gv20376100, TraesCS4D02G160300, TraesCS4A02G162700, TRITD4Av1G113900, TRIDC4AG024580, TuG1812G0400001672, HORVU4Hr1G039410, Bradi1g60357, LOC_Os03g28140, ORUFI03G21190, ONIVA03G22480, OGLUM03G21330, ORGLA03G0192100, OMERI03G18670, BGIOSGA010577, OBART03G20570, OPUNC03G19410, OB03G29750, Sobic.001G345100, Zm00001d029443, Seita.4G162500, AT3G14270, AT4G33240, OBART04G30760, ORGLA04G0271200, OGLUM04G30630,ONIVA04G29940,BGIOSGA017414,LOC_Os04g59540,ORUFI04G32460,OMERI04G25680,OPUNC04G28270,OB04G37780,Zm00001d002940, Sobic.006G280400, Seita.3G002200, TraesCS2A02G559200, TRIDC2AG081800, TuG1812G0200006310, AET2Gv21299600, TraesCS2D02G571300, TRIDC2BG090890, TraesCS2B02G621000, TRITD0Uv1G017220,HORVU2Hr1G127070, HORVU2Hr1G126940,Bradi5g27540,TRIDC7BG037450,TraesCS7B02G225600,TRITD7Bv1G129820,TraesCS7D02G321500, AET7Gv20808200, TraesCS7A02G325000, TRITD7Av1G172560, TRIDC7AG045510, TuG1812G0700003537, HORVU7Hr1G077710, Bradi3g13177, ORUFI08G00390, LOC_Os08g01390, OGLUM08G00410, OBART08G00350, BGIOSGA027837, ONIVA08G00330,OMERI08G00650,OPUNC08G00410,OB08G10420,Sobic.007G004500, Zm00001d049734, Zm00001d035004, Seita.6G005800, ORGLA12G0065400, OBART12G06950, LOC_Os12g13440, BGIOSGA037201, ONIVA12G06810, ORUFI12G07750, OGLUM12G08280, OMERI12G04610, ORUFI09G03780, BGIOSGA030092, LOC_Os09g10650, OGLUM09G04120, OBART09G03340, OMERI09G02610, ONIVA09G03680,OPUNC09G02960,OB09G12130,TRITD3Av1G229250, TraesCS3A02G385500, TRIDC3AG055110, AET3Gv20858900, TraesCS3B02G417700, TRITD3Bv1G215320, TraesCS3D02G378500, TRIDC3BG061700, Bradi2g56547, Seita.2G138800, Seita.5G398600, Sobic.003G373500, Zm00001d042662, AT1G34260, LOC_Os02g06350, OMERI07G07870, ORGLA04G0051200, OBART04G06790, ONIVA04G03320, ORUFI04G07830, LOC_Os04g23420, OMERI04G08980
HSP60	Class III	OPUNC02G17730, OPUNC10G14150, BGIOSGA031559, ONIVA10G17190, OGLUM10G15580, LOC_Os10g37060, ORUFI10G16650, OBART10G15610, OMERI10G12250, OPUNC10G14160, ORUFI02G15280, BGIOSGA028596, ONIVA02G16220, LOC_Os02g22780, OBART02G15030, OGLUM02G15010, OB02G23110, OPUNC02G13400, Sobic.001G530800, Zm00001d051742, Zm00001d027371, Seita.9G569100, TuG1812G0700001730, TRIDC7AG020120, TuG1812G0700004642, AET7Gv20427400, TraesCS7A02G426400, TRIDC7AG059420, TraesCS7D02G164400, TRITD7Av1G229930, TraesCS6B02G391500, TRIDC6BG062660, TRITD7Bv1G026620, TRIDC7BG010160, TraesCS7B02G068100, HORVU7Hr1G034140,

Type	Cluster	ID
HSP60	Class III	TRIDC6AG053750, AET6Gv20885500, Bradi3g11280, AT3G18190, TraesCS4B02G165400, TRIDC4BG025640, TRITD4Bv1G076690, AET4Gv20355900, TraesCS4D02G157300, TRITD4Av1G120450, TraesCS4A02G164900, TRIDC4AG026040, TuG1812S0001848400, HORVU4Hr1G040660, TuG1812G0600000089, TuG1812G0600000077, TRIDC6AG000690, TRITD6Av1G001070, TraesCS6D02G008000, TraesCS6B02G010900, AET6Gv20013600, TRIDC6BG001180, Bradi4g15450, Sobic.008G097966, Sobic.005G163700, Zm00001d007508, Zm00001d052948, Seita.8G160500, ORUFI11G19050, LOC_Os11g36950, OBART11G17860,OGLUM11G17360,ONIVA11G17780,ORGLA11G0149600,BGIOSGA035491,OPUNC11G14670,OB03G44880,OPUNC03G35040,LOC_Os03g59020,BGIOSGA013764, ONIVA03G39390, ORUFI03G39590,OGLUM03G37740,ORGLA03G0346400,OBART03G37980,OMERI11G14380,OMERI03G35140,AT3G03960,TRIDC7AG027450,TRITD7Av1G076790,TraesCS7A02G217200,TuG1812G0700002297, AET7Gv20542300, TraesCS7D02G218500, TRIDC7BG018200, HORVU7Hr1G044070, TRITD7Bv1G051870, TraesCS7B02G124100, AET7Gv20143000, TraesCS7D02G056800, TraesCS7A02G062100, TRIDC7AG006650, TRITD7Av1G015730, TraesCS4A02G427500, TRIDC4AG063360, TRITD4Av1G246670,TuG1812G0700000671,Bradi5g17750,Bradi1g49760,TraesCS1B02G218800, TRITD1Bv1G127970, TRIDC1BG036450,AET1Gv20524000,TraesCS1D02G208500,TRIDC1AG030980,TRITD1Av1G133730,TraesCS1A02G205200,TuG1812G0100002436, HORVU1Hr1G052730, OPUNC02G24240, OPUNC04G18650, OGLUM04G20900, ORUFI04G22570, LOC_Os04g46620, OMERI04G17400, OBART04G20970, ORGLA04G0180300, ONIVA04G19220, BGIOSGA016889, OB04G27990, Zm00001d052056, Zm00001d018349, Sobic.004G326600, Seita.4G052500, Seita.7G190300, AT3G20050, TraesCS6B02G159800, TRIDC6BG023490, TRITD6Bv1G056370, AET6Gv20343300, TraesCS6D02G121300, TRIDC6AG017690, TRITD6Av1G044000, TraesCS6A02G131400, TuG1812G0600000136, HORVU6Hr1G025270, Bradi3g04430, OMERI02G05270, Seita.1G081200, Sobic.004G047200, Zm00001d053857, ORUFI06G27250, LOC_Os06g47320, OGLUM06G26690, ORGLA06G0211000, OBART06G25380, BGIOSGA020645, OGLUM06G26670, ONIVA06G28410, OMERI06G25500, OPUNC06G23080, OB06G33050, AT3G11830, Seita.4G254100, Zm00001d025677, Zm00001d032634, ONIVA05G26500, OGLUM05G26900, ORUFI05G27100, ORGLA05G0218500, OBART05G25300, BGIOSGA020338, LOC_Os05g48290, OMERI05G22570, OPUNC05G22860, OB05G32890, OB05G18880, Zm00001d040688, Zm00001d052882, Zm00001d008369, Zm00001d040257, Sobic.003G112800, Sobic.003G060500, Seita.5G061900, BGIOSGA013131, OMERI03G23650, ONIVA03G27250, OBART03G26220, ORGLA03G0243300, OGLUM03G27110, LOC_Os03g42220, ORUFI03G27170, OPUNC03G23940, OB03G34280, AT5G20890, Bradi2g16500, TraesCS5A02G342600, TuG1812G0500003706, TRIDC5AG050240, TRITD5Av1G189060, TraesCS5B02G341900, TRITD5Bv1G180170, TraesCS5D02G347400, HORVU5Hr1G085530, AET5Gv20789500, TRIDC5BG054220, TRIDC3BG085940, TraesCS3B02G595300, HORVU4Hr1G068820,

Type	Cluster	ID
HSP60	Class III	TRITD4Av1G020190, TRIDC4AG007470, TuG1812G0400002915,TraesCS4A02G054800, TRIDC4BG043560, TRITD4Bv1G150860, TraesCS4B02G250200, AET4Gv20624300, TraesCS4D02G250100,TRIDC7AG050140, TuG1812G0700003893, TRITD7Bv1G151340, TRITD7Av1G193880, TraesCS7A02G358400, TraesCS7B02G269300, TraesCS7D02G364400, AET7Gv20904100, TRIDC7BG043680, HORVU7Hr1G088080, Bradi1g37790, AET6Gv20310900, AET2Gv21275800, TRITD1Bv1G131920, AET1Gv20539800, TraesCS1D02G216600, TraesCS1B02G227600, TRIDC1BG037650,TRITD1Av1G137340,TRIDC1AG032250,TraesCS1A02G213800,TuG1812G0100002528,TRIDC6BG037710,HORVU1Hr1G054530,Bradi3g33860,AT1G24510,OPUNC06G16870, OGLUM06G19740, OMERI06G18990, OBART06G18640, ONIVA06G22310, ORUFI06G19630, LOC_Os06g36700, BGIOSGA021032, OB06G26160, ORGLA06G0154100, Sobic.010G169300,Seita.4G239100,Seita.2G010200,Sobic.002G014400, Zm00001d018694, Zm00001d007960, BGIOSGA024073, ONIVA02G11670,ORUFI02G10480,LOC_Os02g14929,BGIOSGA006793,OBART02G10560,ORGLA02G0095000,OGLUM02G10480,OPUNC02G09410,OMERI02G11420,OB02G19360,AT5G26360,Seita.4G173200,Sobic.001G460500,Zm00001d053553,Zm00001d028183, LOC_Os06g34690,ORGLA06G0146000,ONIVA06G20920,BGIOSGA023051,OBART06G17470,OPUNC06G15830,OGLUM06G18610,ORUFI06G18470,OMERI06G18340,OB06G25210,Bradi1g39090,Bradi2g26420,TraesCS7A02G346200,TuG1812G0700003766,TRITD7Av1G185820, TRIDC7AG048360, TraesCS7D02G332900, AET7Gv20832200, TRIDC7BG039000, TraesCS7B02G237000, TRITD7Bv1G134940, HORVU7Hr1G080310, Bradi3g04130, OBART05G03150, ONIVA05G03140, LOC_Os05g05470, ORUFI05G03300, ORGLA05G0030200, OMERI05G03160, OGLUM05G03200, BGIOSGA018808, OPUNC05G03030, OB05G13100, Zm00001d007192, Sobic.002G423500, Seita.3G039600, AT5G16070, AT3G02530, TRIDC6AG058690, TRITD6Av1G221020, TuG1812G0600004256, HORVU6Hr1G091690, TraesCS6A02G399300, TraesCS6D02G383500, AET6Gv20968000, TRITD6Bv1G221970, TRIDC6BG069470
HSP70	I	TraesCS1B02G139600, TRITD1Bv1G063310, TRIDC1BG021120, TraesCS1D02G121200, AET1Gv20297400, TRITD1Av1G054830, TraesCS1A02G120200, TuG1812G0100001612, TRIDC1AG013330, HORVU1Hr1G027420, Bradi2g33682, Bradi2g33676, TRITD1Bv1G063270, TraesCS1B02G139500, TRIDC1BG021110, AET1Gv20297000, TraesCS1D02G121000, TraesCS1A02G120100, TRITD1Av1G054820, TuG1812G0100001611, HORVU1Hr1G026840, OGLUM05G05300, OBART05G05420, ORGLA05G0048700, BGIOSGA019282, ONIVA05G05880, LOC_Os05g08840, OMERI05G05090, ORUFI05G05720, OPUNC05G05030, OB05G15100, Seita.3G106100, Zm00001d009950, Zm00001d009828, Zm00001d037717, Sobic.009G066900, Seita.3G106000, Zm00001d025053, Zm00001d048458, Zm00001d009948, Zm00001d037700, Sobic.009G067000, ONIVA01G05920, ORGLA01G0048800, OBART01G05250, LOC_Os01g08560, ORUFI01G05580, BGIOSGA002892, OGLUM01G05720, OMERI01G05450,

Type	Cluster	ID
HSP70	I	OPUNC01G05090, OB01G15130, AT1G79920, AT1G79930, TuG1812G0700004936,TRITD7Av1G243980,TraesCS7A02G457100, TRIDC7AG063590, TraesCS7D02G445600, AET7Gv21120800, TRITD7Bv1G193040, TraesCS7B02G359100, TRIDC7BG057410, HORVU7Hr1G105330, Bradi1g32770, Zm00001d014358, Zm00001d036571, Sobic.010G230600, Seita.4G261100, ONIVA06G28060, OBART06G24800, ORUFI06G26710, LOC_Os06g46600, BGIOSGA023469, OGLUM06G26160, OMERI06G25000,OPUNC06G22530,OB06G32560,AT1G11660,TRITD3Bv1G158750,TraesCS3B02G298400, TRIDC3BG044650, TRIDC6BG032760, TRITD6Bv1G087720, HORVU2Hr1G078210, TRIDC2BG052230, TraesCS1B02G206700, TRITD1Bv1G121790, TRITD7Bv1G210270, TuG1812G0300001737, TRIDC7BG064170, TRITD3Av1G061000,TRIDC7AG065910,TRIDC3AG020050,HORVU1Hr1G077070,HORVU1Hr1G040940,HORVU6Hr1G001950,HORVU4Hr1G078830,HORVU2Hr1G036440,TuG1812G0600003050,TRITD6Av1G174000, TraesCS6A02G276700, TRIDC6AG042320, TraesCS6D02G257000, AET6Gv20699100, TRIDC6BG049400, TraesCS6B02G304200, TRITD6Bv1G161760, Bradi3g53100, ONIVA02G32620, BGIOSGA008908, OBART02G29860, ORGLA02G0253900, OGLUM02G30500, LOC_Os02g48110, ORUFI02G31480, OMERI02G28700, OPUNC02G27410, OB02G37040, Zm00001d051609, Zm00001d051607, Sobic.004G263500, Zm00001d017809, Seita.1G293400, AT4G16660, ONIVA04G03110, ORUFI04G04990, BGIOSGA015567, OBART04G04250, ORGLA04G0032700, TuG1812G0200001136, TRIDC2AG013430, TRITD2Av1G030140, Bradi5g05900, TraesCS1B02G163400, TRITD1Bv1G092960, TRIDC1BG026780
HSP70	II	ORGLA05G0118400, OBART05G13770, BGIOSGA018189, OGLUM05G14660, LOC_Os05g30480, ORUFI05G14920, ONIVA05G14310, OMERI05G12270, OPUNC05G11930, OB05G21840, OB06G34100, ORUFI06G16090, ONIVA06G18100, BGIOSGA022924, OBART06G15220, OB01G28800, ORUFI08G06480, BGIOSGA027493, LOC_Os08g09770, ORUFI08G06300, ONIVA08G05680, ORGLA08G0049400, OGLUM08G06010, OMERI05G15090, ORUFI05G18580, LOC_Os05g35400, ONIVA05G18260, BGIOSGA018010, OGLUM05G18420, OBART05G17430, OMERI08G05540, OPUNC05G15340, OB01G36960, TRIDC7BG053030, TraesCS7B02G331400, TRITD7Bv1G181360, AET7Gv21044600, TraesCS7D02G423100, HORVU7Hr1G098810, TRIDC7BG052970, TraesCS7B02G330900, TRITD7Bv1G181250, AET7Gv21043500, TraesCS7D02G422600, TRIDC7AG060130, TraesCS7A02G430600, TRITD7Av1G232280, TraesCS5A02G298700, TRITD5Av1G172440, TRIDC5AG044640, TraesCS5D02G305500, AET5Gv20692700, TRITD5Bv1G163290, TraesCS5B02G298000, TRIDC5BG048500, HORVU5Hr1G078400, Sobic.003G178101, OBART12G16290, Sobic.003G378700, Seita.5G187000, Seita.5G187500, Seita.3G341900, LOC_Os01g33360, BGIOSGA003641, ORUFI01G19810, ONIVA01G19750, BGIOSGA003640, OMERI01G16660, OGLUM01G21090, ORGLA01G0145900, ORGLA11G0065000, OBART01G17830, OB01G27370, TRITD2Bv1G038020, TraesCS2B02G125400, TRIDC2BG015910, TraesCS2A02G107900, TRITD2Av1G029060,

Type	Cluster	ID
HSP70	II	TraesCS2D02G108000, TRIDC2AG078660, TRITD2Av1G288840, TRIDC2AG078650, TRITD2Av1G288880, TraesCS1B02G333200, TRIDC1BG054010, TRIDC2AG078670, TRITD2Av1G288990, TuG1812G0200006178, TraesCS2A02G585600, AET2Gv21240500, TRITD2Bv1G257990, TraesCS2D02G600900, AET2Gv21241200, TRITD2Bv1G257820, TRIDC2BG086730, TraesCS1D02G452500, AET1Gv21047200, TRITD2Bv1G257810, TRIDC2BG086830, HORVU2Hr1G122760, HORVU2Hr1G122800, Bradi2g06050, Bradi4g28250, Seita.3G012300, ORUFI02G01160, OGLUM02G01040, BGIOSGA007251, LOC_Os02g02410, ONIVA01G37420, OBART02G01170, OMERI02G00580, ORGLA02G0010600, OPUNC02G00610, OB02G10970, Seita.1G117600, Zm00001d018953, Zm00001d018975, Zm00001d047525, TuG1812S0001400100, Zm00001d054043, Zm00001d014993, Sobic.004G011700, Bradi3g01477, TRIDC6BG007400, AET6Gv20108600, TRITD6Bv1G012740, TraesCS6D02G049100, TuG1812S0001602300, TraesCS6A02G042600, TraesCS6B02G058300, TRITD6Av1G008390, TRIDC6AG005090, AT5G42020, AT5G28540, Zm00001d041164, Zm00001d021055, BGIOSGA013428, LOC_Os03g50250, ONIVA03G32850, ORGLA03G0292000, OBART03G31550, OGLUM03G31800, ORUFI03G32850, OMERI03G28170, OPUNC03G28740, OB03G38590, Sobic.001G118600, OB07G16700, Seita.9G120300, AT1G09080
HSP70	II-like	TraesCS4D02G338300, AET4Gv20801800, TRIDC4BG057620, TRITD4Bv1G192700, TraesCS5A02G511700, TRITD5Av1G244310, TuG1812G0500005231, HORVU4Hr1G084190, HORVU4Hr1G084180, TRIDC5AG073030, TRITD5Av1G244440, TuG1812G0500005233, TraesCS5A02G511800, TraesCS4B02G342600, HORVU4Hr1G084200, TraesCS4D02G338600, AET4Gv20802100, Bradi1g75681, TRIDC7BG059080, TRITD7Bv1G196770, TraesCS7B02G369100, TraesCS7D02G455300, TraesCS7D02G455000, HORVU7Hr1G107190, ORGLA05G0242300, OBART05G27860, OMERI05G23750, LOC_Os05g51360, ORUFI05G29820, ONIVA05G29600, OGLUM05G29320, ONIVA03G22380, BGIOSGA021688, ORUFI06G07350, LOC_Os06g10990, OGLUM06G07560, OMERI06G08350, OBART06G07170, ORGLA06G0067200, OBART06G07180, OB02G41600, Zm00001d025554, Zm00001d025565, Zm00001d025561, Zm00001d025556, Zm00001d026467, Sobic.009G254800, TRITD5Bv1G027870, TraesCS5B02G065900, TRIDC5BG010730, TRIDC5BG010720, AET5Gv20172300, TraesCS5D02G070100, HORVU5Hr1G014490, Seita.2G003700, Sobic.002G008000, Bradi4g43170, Seita.7G325600, Seita.6G232400, Sobic.008G038700, ORGLA12G0031300, OGLUM12G03960, OBART12G03290, BGIOSGA037007, LOC_Os12g05760, OMERI03G27420, OPUNC12G03060, OPUNC12G03090, OB12G13250, Bradi4g39820, Bradi4g39850, OB03G20920, TuG1812G0400003064, TRIDC4AG005550, AET2Gv21257700, TRITD5Bv1G240440, TraesCS5B02G517800, TraesCS6D02G339600, TraesCS6D02G339700, TraesCS6D02G339800, HORVU6Hr1G094830, HORVU6Hr1G009570

Type	Cluster	ID
HSP70	III	TraesCS1A02G295600, TRITD1Av1G180370, TRIDC1AG043890, TuG1812G0100003356, AET2Gv21290700, TraesCS2D02G575200, TraesCSU02G116400, AET4Gv20882800, TraesCS4B02G397600, TRITD4Bv1G207630, TRIDC4BG063730, TRIDC5AG069720, TuG1812G0500005040, HORVU4Hr1G089090, HORVU3Hr1G073230, HORVU0Hr1G006930, AET3Gv20701400, TraesCS3D02G296700, TRITD3Av1G191970, TraesCS3A02G305100, TuG1812S0002607000, TRIDC3AG043960, TraesCS3B02G331400, TRITD3Bv1G174600, TRIDC3BG049910, Bradi3g57450, OB03G11040, BGIOSGA005513, OBART02G34330, LOC_Os02g53420, ORGLA02G0297200, OGLUM02G35500, ONIVA02G36410, ORUFI02G35880, OMERI02G32610, OPUNC02G31690, OB02G41190, LOC_Os03g02260, ORUFI03G00840, ORGLA03G0008500, BGIOSGA011582, ONIVA03G01190, OBART03G01050, OGLUM03G01030, OMERI03G00920, OPUNC03G00960, Bradi1g77637, Zm00001d013842, Zm00001d024739, Sobic.001G193500, Seita.9G191200, Seita.2G218100, AT5G09590, AT4G32208, AT4G37910, HORVU3Hr1G030980, HORVU4Hr1G012460, OB03G11030, HORVU5Hr1G073730, TRITD5Av1G160450, TuG1812G0500003004, TRIDC5AG040630, TraesCS5A02G268100, TraesCS5B02G267900, TRITD5Bv1G152350, TRIDC5BG044380, TraesCS5D02G276100, AET5Gv20629700, Bradi4g33878, OGLUM09G15530, OBART09G14910, ONIVA09G15520, BGIOSGA029532, OMERI09G11580, OPUNC09G13350, ORUFI09G16070, LOC_Os09g31486, OB09G21470, Sobic.002G249800, Zm00001d006036, Seita.2G255900, Zm00001d020956, TRIDC5BG019680, TraesCS5B02G111200, TRITD5Bv1G058760, TraesCS5D02G117600, AET5Gv20312000, TRIDC5AG016610, TraesCS5A02G106200, TRITD5Av1G065550, TuG1812G0500001472, HORVU5Hr1G032650, Bradi4g39470, LOC_Os12g14070, ORUFI12G08020, ONIVA12G07210, BGIOSGA037214, ORGLA12G0068700, OGLUM12G08560, OBART12G07220, OMERI12G04830, OPUNC12G06780, OB12G16680, Zm00001d023802, Zm00001d041119, Sobic.008G088200, Seita.3G086700, TRITD1Av1G067490, TuG1812G0100001662, TraesCS1A02G133100, TRIDC1AG019180, TraesCS1B02G151300, TRITD1Bv1G078090, TRIDC1BG023520, TraesCS1D02G131800, AET1Gv20328300, HORVU1Hr1G030790, Bradi2g30560, LOC_Os05g23740, OPUNC05G08990, OGLUM05G11140, ORUFI05G11620, ONIVA05G11020, ORGLA05G0093100, OBART05G10750, BGIOSGA019575, OB05G19340, Sobic.001G129000, Zm00001d033591, Zm00001d013507, Seita.3G250300, AT4G24280, AT5G49910, Zm00001d020199, TRITD5Bv1G244830, Zm00001d019061, ONIVA01G21500, Sobic.006G045200
HSP70	IV	OBART01G27060, ONIVA01G31030, ORGLA01G0222300, OGLUM01G31000, LOC_Os01g49430, ORUFI01G30100, BGIOSGA000999, OMERI01G24900, OPUNC01G27050, OB01G36210, TRITD3Bv1G155490, TRIDC3BG044340, TraesCS3B02G296300, TraesCS3D02G262800, TRITD3Av1G175010, TraesCS3A02G262900, TuG1812G0300003091, Bradi2g46937

Type	Cluster	ID
HSP70	V	LOC_Os03g16880, LOC_Os11g08470, BGIOSGA034363, OGLUM11G05290, LOC_Os11g08445, BGIOSGA034960, OPUNC11G05220, OBART11G05440, ORUFI11G05270, ORUFI11G05320, ONIVA11G05820, BGIOSGA034959, LOC_Os11g08440, OB11G14460, OGLUM11G05270, ORUFI11G05290, OBART11G05430, BGIOSGA034961, ONIVA11G05830, ORUFI11G05280, OGLUM11G05260, ONIVA11G05840, BGIOSGA034364, BGIOSGA034362, LOC_Os11g08460, OGLUM11G05280, ORUFI11G05310, OPUNC11G05230, OB11G14490, OB11G14470, OB11G14480, Sobic.001G419801, Sobic.001G419100, Sobic.001G419000, Zm00001d028639, Seita.4G060500, Sobic.001G419700, Sobic.001G419200, Sobic.001G419500, Sobic.001G419600, Sobic.001G419300, Sobic.001G419900, Sobic.001G419400, TRIDC1BG061620, TRIDC7BG038920, AET2Gv20298000, TRIDC6AG017160, TRIDC1BG057710, TRIDC2BG022220, Zm00001d020058, TRIDC2BG029110, AT1G16030, TraesCS4A02G098600, TRITD4Av1G045640, TuG1812G0400002453, TRIDC4AG013450, AET4Gv20525900, TraesCS4D02G206600, HORVU4Hr1G059260, TraesCS4B02G205700, TRITD4Bv1G120830, TRIDC4BG036910, Bradi1g66470, ORGLA03G0120400, OGLUM03G12460, ONIVA01G18750, BGIOSGA011002, LOC_Os03g16920, ORUFI03G13100, OBART03G12430, OMERI03G11660, OPUNC03G12020, OB03G22520, Seita.9G451500, Zm00001d047799, Sobic.001G418600, ONIVA11G05850, Seita.5G376100, Sobic.003G350700, Zm00001d012420, Zm00001d042922, Sobic.006G055600, AET3Gv20805300, TraesCS3D02G352400, TraesCS3D02G351900, TRITD3Bv1G202480, TRITD3Bv1G202720, TraesCS3B02G390800, TRIDC3BG058180, TraesCS3B02G390700, TRITD3Bv1G202470, TRIDC3BG058250, TRITD3Bv1G202710, TuG1812S0002645100, TRIDC3AG051540, HORVU3Hr1G086500, Bradi2g54570, TRIDC1AG042600, TraesCS1A02G285000, TuG1812G0100003247, TRITD1Av1G176390, TraesCS1D02G284000, AET1Gv20682100, TRIDC1BG048340, TraesCS1B02G294300, TRITD1Bv1G162470, HORVU1Hr1G072220,Bradi2g23250,ONIVA05G20120,OGLUM05G 20340, BGIOSGA017916, LOC_Os05g38530, ORUFI05G20460, OMERI05G16790, ORGLA05G0167000, OBART05G19250, OPUNC05G16870,OB05G26830,Seita.3G216900,Sobic.009G163900, Zm00001d010529, OB01G45440, OPUNC02G03140, OPUNC01G35580, AET6Gv21008500, TraesCS6D02G402500, Seita.8G225900, Zm00001d007706, Zm00001d048687, Seita.8G225000, BGIOSGA012332, Bradi5g17520, OMERI01G33810, LOC_Os01g62290, BGIOSGA004771, OGLUM01G40600, ORGLA01G0305400, OBART01G36690, ONIVA01G41820, ORUFI09G15530, TRITD4Av1G045040, TRIDC4AG013340, TraesCS4A02G097900, TuG1812G0400002454, TraesCS4D02G207500, AET4Gv20528300, TraesCS4B02G206700, TRIDC4BG037050, TRITD4Bv1G126530, HORVU4Hr1G059390, Bradi1g66590, ONIVA11G13600, BGIOSGA012333, OMERI03G11590, LOC_Os03g16860, OBART03G12360, ORGLA03G0119700, ORUFI03G13030, OGLUM03G12380, OPUNC03G11940, OB03G22450, Zm00001d028630, Sobic.001G420100, Seita.9G451900, AT3G12580, AT5G02490,

Type	Cluster	ID
HSP70	V	AT1G56410, AT3G09440, AT5G02500, TraesCS4A02G175100, TRITD4Av1G138530, TRIDC4AG028230, TRIDC4BG022350,TRITD4Bv1G062200, TraesCS4B02G142400, TuG1812G0400001495, AET4Gv20300300, TraesCS4D02G140800, AET7Gv20781800, ONIVA11G23070, BGIOSGA035738, ORUFI11G25440, OBART11G23560, ORGLA11G0189300, OGLUM03G10270, LOC_Os11g47760, BGIOSGA035736, OPUNC11G19900, OMERI08G15730, ORGLA12G0142900, ORGLA12G0176900, OBART12G16280, BGIOSGA037626, ORUFI12G18100, ONIVA02G21990, LOC_Os12g38180, OGLUM12G17850, OGLUM12G17590, BGIOSGA010330, OB11G27940, TRITD3Bv1G209030, OPUNC12G14780, OB12G23160, TRIDC5BG073650, TRITD5Av1G232950, TRIDC5AG068400, TuG1812G0500004901, TraesCS5D02G492900, TraesCS5B02G492500, TRITD5Bv1G232320, TraesCS5A02G479300, HORVU5Hr1G113180, AET5Gv21108900, Bradi1g03720, OPUNC03G36190, BGIOSGA013836, OMERI03G35920, LOC_Os03g60620, ONIVA03G40770, OGLUM03G38960, ORUFI03G40780, OB03G46070, TRIDC1BG004360, ORGLA03G0358700, OBART03G39090, Seita.9G033500, Zm00001d041550, Sobic.008G136000, Seita.3G327900, Zm00001d030725, Bradi4g04220, TuG1812G0500000901, TRIDC5AG012460, TRITD5Av1G040700, TraesCS5A02G078000, AET5Gv20225300, TraesCS5D02G093900, TRIDC5BG014390, TraesCS5B02G087700, TRITD5Bv1G039950, TraesCS5B02G129700, TRIDC5BG022950, TRITD5Bv1G080790, HORVU5Hr1G021310, HORVU5Hr1G021300, TRITD2Bv1G178040, TRIDC5AG004990, HORVU6Hr1G081460, TraesCS2B02G374700, TRIDC2BG054830, TRITD6Av1G202080, TraesCS6A02G337100, TraesCS6B02G367800, TRIDC6AG050480, AET6Gv20830600, TraesCS6D02G317700, TRIDC6BG059090, TRITD6Bv1G197040
HSP70	V-like	TRITD6Av1G204460, TRIDC6AG051240, TraesCS6A02G342200, TuG1812G0600003717, TraesCS6B02G374500, TRIDC6BG060070, TraesCS6A02G342600, TraesCS6B02G374400, TRITD6Bv1G199910, TraesCS6D02G322700, AET6Gv20847000, HORVU6Hr1G082510, TuG1812S0003481000, TRIDC6BG060110, HORVU6Hr1G082600, TraesCS6A02G342500, TRIDC6AG051280, TraesCS2B02G535000, TRITD2Bv1G236970, TRIDC2BG077170, AET2Gv21114000, TRIDC2AG071450, TraesCS2A02G506900, TRITD2Av1G273670, TuG1812G0200005508, HORVU2Hr1G112630, TraesCS2A02G507000, TRIDC2AG071440, TuG1812G0200005509, TraesCS4A02G098200, TRITD4Av1G045270, TRIDC4AG013400, TuG1812G0400002458, TRITD4Bv1G126420, TRIDC4BG037010, TraesCS4B02G206300, TraesCS4D02G207100, AET4Gv20526800, Bradi1g66527, Bradi1g66520, TRIDC4BG037020, AET4Gv20527500, TRITD4Bv1G126430, TraesCS4B02G206400, HORVU4Hr1G059210, TraesCS4A02G098100, TRITD4Av1G045220, TRIDC4AG013380, TuG1812G0400002457, Bradi1g66540, Bradi1g66550, TRIDC6BG060090, TRITD6Bv1G199980, TraesCS6B02G374600, TRIDC6BG060100, TRIDC6AG051270, TraesCS6A02G342400, TRITD6Av1G204530, AET6Gv20847200, TraesCS6D02G322800, TuG1812G0600003738,

Type	Cluster	ID
HSP70	V-like	TRITD6Av1G204720, TraesCS6A02G342900, TuG1812G0600003739, HORVU6Hr1G082550, HORVU6Hr1G082530, TRIDC6AG051260, TuG1812G0600003716, TRIDC6BG060080, TRITD6Bv1G199960, Bradi1g66561, OBART03G12390, ORGLA03G0120000, OGLUM03G12410, OMERI03G11610, OPUNC03G11970, OB03G22480, BGIOSGA011005, ONIVA11G13630, ORUFI03G13060
HSP70	VI	TraesCS4B02G243400, TRITD4Bv1G147020, TRIDC4BG042680, TraesCS4D02G243000, AET4Gv20606300, TRITD4Av1G023620, TraesCS4A02G066100, TRIDC4AG008500, TuG1812G0400002862, Bradi1g69700, Sobic.001G454000, Zm00001d048073, Seita.9G488400, LOC_Os03g11910, ORUFI03G08690, OMERI03G08290, OGLUM03G08700, ONIVA03G09410, BGIOSGA011204, OBART03G08570, ORGLA03G0083000, OPUNC03G08250, OPUNC03G08260, OB03G18890, AT2G32120
HSP90	Group 1	HORVU5Hr1G072420, TRITD5Av1G157760, TraesCS5A02G260600, TRIDC5AG019020, TRITD5Bv1G146740, TraesCS5D02G268000, AET5Gv20611500, TraesCS5B02G258900, TRIDC5BG042990, TuG1812G0500002709, TRITD5Av1G149830, AT5G56000, AT5G56010, AT5G56030, HORVU7Hr1G049370, TraesCS7A02G242200, TRITD7Av1G089150, TRITD7Bv1G071080, TRIDC7BG023680, TraesCS7B02G149200, TRIDC7AG030920, TuG1812G0700002610, TraesCS7D02G241100, AET7Gv20594700, Bradi3g39590, Bradi3g39620, Bradi3g39630, Seita.6G200300, Seita.6G200200, Seita.6G200000, Zm00001d031332, Sobic.007G216300, Zm00001d052855
HSP90	Group 2	LOC_Os09g36420, OGLUM09G18210, ORUFI09G19060, OMERI09G13130, OBART09G17650, ORGLA09G0138700, OPUNC09G16040, ONIVA09G18690, BGIOSGA031149, OB08G26460, OB08G26470, OB08G26480, OB08G27440, LOC_Os08g39140, ONIVA08G21710, OGLUM08G20570, BGIOSGA026730, ORUFI08G21770, OBART08G19470, ORGLA08G0171400, OMERI08G16110, OPUNC08G17530, ORUFI09G15300, BGIOSGA029569, OMERI09G11040, ORUFI09G19020, OPUNC09G12760, ORGLA09G0109400, OMERI09G11050, LOC_Os09g30418, OBART09G14250, ONIVA09G14810, BGIOSGA029571, ORUFI09G15280, LOC_Os09g30412, OGLUM09G15170, OPUNC09G12730, Sobic.002G243200, Sobic.002G243500, Zm00001d006008, Zm00001d020898, OGLUM04G00340, LOC_Os04g01740, ORGLA04G0004100, OBART04G00370, BGIOSGA015767, ORUFI04G00430, ONIVA04G00360, OMERI04G00370, OPUNC04G00450, Sobic.006G005600, Zm00001d024903, Seita.7G009200, TRITD0Uv1G009860, TRIDC2AG003590, TraesCS2A02G033700, TraesCS2D02G033200, TRIDC2BG004160, AET3Gv21097100, TRITD2Bv1G010840, TraesCS2B02G047400, Bradi5g02037, AT5G52640
HSP90	Group 3	TuG1812S0002675000, TRITD5Av1G154160, TRIDC5AG039220, TraesCS5A02G251000, TRIDC5BG041580, TRITD5Bv1G142670, TraesCS5B02G249000, HORVU5Hr1G070720, AET5Gv20592500, TraesCS5D02G258900, Bradi4g32941, Seita.2G241600, Seita.2G071900, Zm00001d020827, ONIVA09G14300,

Type	Cluster	ID
HSP90	Group 3	BGIOSGA029594, OGLUM09G14360, ORUFI09G14760, OBART09G13790, ORGLA09G0105200, LOC_Os09g29840, OPUNC09G12270, BGIOSGA026764, ORUFI08G20930, LOC_Os08g38086, OGLUM08G19750, ONIVA12G18410, ORGLA08G0163400, OBART08G18770, OPUNC08G16710, OB08G25870, Bradi3g38897, Zm00001d052809, Sobic.007G224100, Seita.6G191100, AT2G04030
HSP90	Group 4	TRITD5Av1G058920, TraesCS5A02G101900, TRIDC5AG015710, TuG1812G0500001104, TraesCS5B02G106300, TRITD5Bv1G052150, TRIDC5BG017740, TraesCS5D02G113700, AET5Gv20269900, HORVU5Hr1G027910, Bradi4g06370, ONIVA05G29350, BGIOSGA036094, OBART12G13670, LOC_Os12g32986, ORGLA12G0120500, ORUFI12G15000, OGLUM12G15350, OMERI12G09640, OPUNC12G12340, OB12G21340, Zm00001d035285, Zm00001d041719, Sobic.008G111600, Seita.3G351500, AT3G07770
HSP90	Group 5	AET7Gv21285700, TRITD0Uv1G065770, TraesCS7B02G446900, TraesCS7D02G517800, TraesCS7A02G529900, TRITD7Av1G270370, TuG1812S0001921700, TRIDC7AG074030, HORVU7Hr1G117000, HORVU1Hr1G019830, HORVU7Hr1G055460, Bradi1g30130, Zm00001d036401, Sobic.010G267400, Zm00001d014792, Seita.4G281500, OB06G35350, OB06G35360, ONIVA06G30100, OMERI06G27590, OGLUM06G28960, LOC_Os06g50300, ORGLA06G0232400, ORUFI06G29490, OBART06G27530, BGIOSGA020527, OPUNC06G25200, AT4G24190
